# Chromosome-level *Dinobdella ferox* genome provided a molecular model for its specific parasitism

**DOI:** 10.1186/s13071-023-05837-7

**Published:** 2023-09-11

**Authors:** Jiang-Wei Gao, Jian-Wei Sun, Xiang-Rong Tong, Hao Wang, Qing-Mei Hu, Yan-Ru Cao, Zhan-Han Zhou, Zi-Chao Liu

**Affiliations:** 1 Engineering Research Center for Exploitation and Utilization of Leech Resources in Universities of Yunnan Province, School of Agriculture & Life Sciences, Kunming University, Kunming, China; 2 Department of Medical Ultrasonography, Fifth Affiliated Hospital, Kunming Medical University, Gejiu, China; 3 School of XJTLU Wisdom Lake Academy of Pharmacy, Xi’an Jiaotong-Liverpool University, Suzhou, China

**Keywords:** Leech, *Dinobdella ferox*, Chromosome-level genome, Parasitism modeling, Interspecific molecular interaction, Parasitology

## Abstract

**Background:**

*Dinobdella ferox* is the most frequently reported leech species parasitizing the mammalian nasal cavity. However, the molecular mechanism of this special parasitic behavior has remained largely unknown.

**Methods:**

PacBio long-read sequencing, next-generation sequencing (NGS), and Hi-C sequencing were employed in this study to generate a novel genome of *D. ferox*, which was annotated with strong certainty using bioinformatics methods. The phylogenetic and genomic alterations of *D. ferox* were then studied extensively alongside the genomes of other closely related species. The obligatory parasitism mechanism of *D. ferox* was investigated using RNA-seq and proteomics data.

**Results:**

PacBio long-read sequencing and NGS yielded an assembly of 228 Mb and contig N50 of 2.16 Mb. Along Hi-C sequencing, 96% of the sequences were anchored to nine linkage groups and a high-quality chromosome-level genome was generated. The completed genome included 19,242 protein-coding genes. For elucidating the molecular mechanism of nasal parasitism, transcriptome data were acquired from the digestive tract and front/rear ends of *D. ferox*. Examining secretory proteins in *D. ferox* saliva helped to identify intimate connections between these proteins and membrane proteins in nasal epithelial cells. These interacting proteins played important roles in extracellular matrix (ECM)–receptor interaction, tight junction, focal adhesion, and adherens junction. The interaction between *D. ferox* and mammalian nasal epithelial cells included three major steps of pattern recognition, mucin connection and breakdown, and repair of ECM. The remodeling of ECM between epithelial cells of the nasal mucosa and epithelial cells of *D. ferox* may produce a stable adhesion environment for parasitism.

**Conclusions:**

Our study represents the first-ever attempt to propose a molecular model for specific parasitism. This molecular model may serve as a practical reference for parasitism models of other species and a theoretical foundation for a molecular process of parasitism.

**Graphical abstract:**

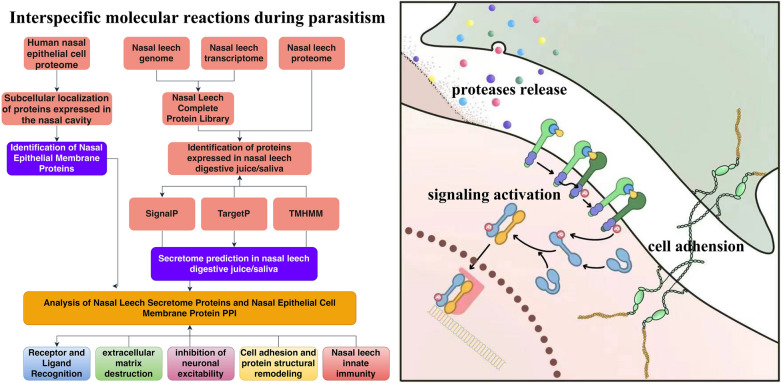

**Supplementary Information:**

The online version contains supplementary material available at 10.1186/s13071-023-05837-7.

## Background

Family Praobdellidae have a life cycle of parasitizing on mammalian mucous membranes [56, 57]. Praobdellid leeches primarily parasitize the upper airway in mammals, and the nasal cavity is known as the most prevalent site of infection [34, 55]. Leech infestation as a foreign body in the human respiratory system has been reported predominantly in Asia, the Mediterranean, and Africa [15, 17, 46]. Based upon the locations of praobdellid species and the photos from previous studies, *Dinobdella ferox, Limnatis nilotica*, and *Myxobdella africana* were the most common species parasitizing humans [6, 42]. Prevalent in South, Southeast, and East Asia, *D. ferox* is the most frequently examined praobdellid leech. Numerous case studies of *D. ferox* infestations in the upper airway have been reported [1, 56].

In the early 2000s, researchers examined the host specificity of *D. ferox*, its development curve during the parasitic phase, and the behavioral inclination of free-living individuals before and after the parasitic period [13, 34]. It was well known that juvenile *D. ferox* could only parasitize mammals and not avian or amphibian hosts, and its growth curve was correlated with host size. Within a month, its mean parasitizing biomass expanded more rapidly in rabbits than in rats, and host mortality was indeed caused by its infestation. Furthermore, there was a notable inclination to dwell at the water surface before parasitizing, and it remained sensitive to airflow and vibration from hosts [34, 48]. In contrast, after parasitizing, leeches preferred to settle at the bottom and remain unresponsive to host cues.

Despite exciting discoveries on its behavior, life history, and host specialization, the molecular processes of its specificity to the parasitic mammalian nasal cavity remain elusive. Thus genome-wide analysis of *D. ferox* helped us to gain insights into the above puzzle. The high-quality chromosome-level genome of *D. ferox* was composed of 228 Mb, 335 contigs, and nine chromosomes. This genome facilitated a deeper understanding of the genetic basis of *D. ferox* parasitism, life history, and reproductive transition.

## Methods

### Sample collection

Samples of *D. ferox* were collected from Nankang Village, Lujiang Town, Longyang District, and Baoshan City in Yunnan Province, China (Global Positioning System [GPS] coordinates: 98°46′6.01″ E, 24°50′3.01″ N). The detailed sampling procedure was as follows: The hosts for nasal leeches in this research were cattle. Cattle were exposed to sunlight for 1–2 h, making them thirsty. Subsequently, a bucket with some water was placed in front of the cattle, but they were not allowed to drink from it. At this point, the nasal leeches also required water, prompting them to extend the anterior part of their body out of the nostril. A hemostatic forceps was then used to firmly grasp and extract the leech. Leeches were brought back to the laboratory alive. Tissues of different parts were separated by ophthalmic scissors and immediately placed in liquid nitrogen for DNA/RNA/protein extraction.

### DNA and RNA extraction

Genomic DNA was extracted by the classic phenol–chloroform method and total RNA extracted with TRIzol Reagent (Invitrogen). The quality and quantity of extracted DNA/RNA were assessed with an Agilent 2100 Bioanalyzer (Agilent Technologies, Inc., Santa Clara, CA, USA), and integrity was further evaluated on agarose gel after a stain of ethidium bromide. The resulting DNA/RNA samples were stored at −80 °C until subsequent library construction and genomic/transcriptomic sequencing. All samples were collected by our own staff.

### Library construction and sequencing

PacBio libraries were constructed and sequenced on the PacBio sequencing platform (https://www.pacb.com/products-and-services/consumables/). For Hi-C sequencing, genomic DNA samples were pretreated according to Rao et al. [61]. Under the adsorption of avidin magnetic beads, biotinized DNA was captured and DNA fragments were end-repaired, adapter-ligated, polymerase chain reaction (PCR)-amplified, and purified in strict accordance with the Illumina Hi-C library protocol. Then the quality of the library was tested according to the standard steps of library quality control. A Qubit 2.0 fluorometer was utilized for preliminary quantification and the library was diluted to 1 ng/μl. The integrity of library DNA fragments and size of insert were detected by the Agilent 2100 system. Then, quantitative PCR (qPCR) was employed to detect the effective concentration of the library for accurate quantification (effective concentration > 2 nM). After library inspection, different libraries were pooled according to the requirements for effective concentration and target data volume. Later, Illumina PE150 sequencing was performed. For library construction of second-generation Illumina sequencing, the following procedures were implemented (https://www.illumina.com/techniques/sequencing/ngs-library-prep.html).

### Quality control of sequencing data

For the Illumina next-generation sequencing (NGS) data, low-quality reads, linker sequences, and repetitive sequences were removed using Trimmomatic [8] and the FASTX-Toolkit [22]. Finally, data quality was evaluated using FastQC [2]. For long PacBio reads, the mean quality for each read was calculated, and only reads longer than 1 kilobase (kb) with mean quality ≥ 7 were retained. For Hi-C data, the sequences with linkers were removed using HiC-Pro [63], and the sequences with N > 10% were retained. The number of low-quality (< 5) bases contained in single-end sequencing reads exceeded 20% of read length and paired reads for removal.

### Estimation of genomic size

Genomic size was estimated by the k-mer method with short-insert library reads. Here, 17-mer was selected for k-mer analysis, and genomic size (Mb) was estimated with the following formula: G = K_num_/K_depth_, where K_num_ and K_depth_ denote the total number and peak depth of 17-mers, respectively.

### Genomic assembly and chromosomal construction

PacBio's third-generation data were corrected to obtain higher accuracy. Clean data were processed with Canu [31] software and then assembled using SMARTdenovo [38] based upon the corrected data. Then NGS data were subjected to triple rounds of calibration by Pilon [71]. Finally, filtered Hi-C reads were aligned to the assembled genome and then anchored to chromosomes using ALLHiC (0.9.8).

### Genome assembly quality assessment

Quality assessment of genomic assemblies for *D. ferox* was performed using BUSCO (Benchmarking Universal Single-Copy Orthologs: http://busco.ezlab.org/) [62] and CEGMA (Core Eukaryotic Genes Mapping Approach: http://korflab.ucdavis.edu/datasets/cegma/) [50]. BUSCO utilized a single-copy orthologous gene library along with tBLASTn, Augustus, and HMMER and other software tools for evaluating the integrity of assembled genomes. CEGMA was adopted for selecting conserved genes (458 genes) in six eukaryotic model organisms for constructing a core gene library along with tBLASTn, GeneWise, and geneid software tools for evaluating the integrity of the assembled genome.

### Genomic annotation

Genomic annotation included the three major aspects of repetitive sequence annotation, gene annotation, and non-coding RNA (ncRNA) annotation. Two annotation methods were used for repetitive sequence annotation, homologous sequence alignment and de novo prediction. The homologous sequence alignment method was based upon the repeat sequence database (http://www.girinst.org/repbase/), using RepeatMasker [11] and RepeatProteinMask software for identifying repeat sequences similar to each other. Ab initio prediction was first performed using LTR_FINDER [74], RepeatScout [59], RepeatModeler [21], and other software to establish the de novo repeat sequence library, and then it was predicted using RepeatMasker software. Protein-coding genes were annotated through three combined approaches of de novo prediction, homology-based annotation, and/or transcript-based annotation from the repeat-masked genome. For de novo prediction, Augustus (v.3.2.1) [68] and GENSCAN (v.1.0) [24] were employed. For homology-based annotation, protein sequences of related species were downloaded from the National Center for Biotechnology (NCBI) database. Then, the Basic Local Alignment Search Tool (BLAST) and GeneWise were employed for predicting genetic structures. The resulting annotated gene set was compared with Swiss-Prot (http://www.uniprot.org/), Nr (http://www.ncbi.nlm.nih.gov/protein), Pfam (http://pfam.xfam.org), Kyoto Encyclopedia of Genes and Genomes (KEGG) (http://www.genome.jp/kegg/), InterPro (https://www.ebi.ac.uk/interpro/), and other databases to obtain gene functional information. Also, tRNAscan-SE [39] software was utilized for locating transfer RNA (tRNA) sequences in the genome. Since ribosomal RNA (rRNA) is highly conserved, rRNA sequences of closely related species may be selected as reference sequences and rRNA in the genome identified by BLAST alignment. The covariance model of the Rfam family was utilized and INFERNAL (INFERence of RNA ALignment) [47] software in Rfam was used for predicting microRNA (miRNA) and small nuclear RNA (snRNA) sequence information in the genome.

### Identification of orthologous genes and phylogenetic tree construction

Orthologs were identified along with nine closely related species. And the similarity relationship between protein sequences of all species was obtained using all-versus-all BlastP, and the results were clustered with OrthoMCL [36] software. Maximum likelihood (ML) phylogenetic trees based upon multiple sequence alignments were constructed using RAxML [66].

### Estimation of gene family expansion and contraction

Expansion and contraction of the gene family was determined using CAFÉ software (v.3.1) [16]. The phylogenetic tree and divergence time of previous steps were imported into CAFÉ to infer the changes in gene family size using a probability model.

### Gene expression profiling

RNA sequencing (RNA-seq) data were acquired for the digestive tract and front/back body ends. Raw reads were filtered and the remaining high-quality reads aligned to the assembled genome using HISAT2 [52]. The transcripts were assembled with StringTie [53], and gene expression values were analyzed using Salmon software [51].

### Protein extraction

First, the digestive tract tissues of nasal leeches were collected and the microbes in the digestive tract secretions were removed via differential centrifugation. Subsequently, protein extraction was performed using a well-established method from previous research. Exogenous protein contamination could not be completely avoided during sampling. In the following steps, a highly reliable nasal leech protein database was obtained through genomic and transcriptomic data, which was used to filter out non-leech proteins during proteomics analysis by searching against the database.

### Mass spectrometry (protein)

The data-dependent mode of the Q Exactive Orbitrap Mass Spectrometer (Thermo Finnigan LLC, San Jose, CA, USA) was selected for automatic transition between full-scan mass spectroscopy (MS) and tandem MS (MS/MS collection. Full-scan MS spectra (m/z 350–1300) were acquired with a resolution of 70,000 (m/z 200) after accumulating ions to a 3106 target value based upon predicted automated gain control from the previous full scan. Dynamic exclusion time was set at 18 s. The MS2 scanning technique selected and fragmented the 10 most intense multiply charged ions (z = 2) successively using higher-energy collisional dissociation (HCD) with a fixed injection duration of 60 ms and a resolution of 17,500 (m/z 200). The spray voltage was set to 2 kV. There was no sheath or auxiliary gas flow. The capillary was heated to 250 °C, the normalized HCD collision energy was 27 eV, and the underfill ratio was 0.1%. The ion selection threshold for MS/MS was set at 1 × 10^5^ counts.

### Prediction of leech secretome

A protein library was generated with RNA-seq and proteomics employed for assessing the protein composition of saliva and digestive fluid. Then the secretome was compiled with SignalP [54], TargetP [18], and TMHMM for identifying the proteins secreted from saliva and digestive fluid [32].

### Prediction of human nasal epithelial cell membrane protein

Proteomics data of human nasal epithelium were downloaded from publicly available databases. Then subcellular localization data of these proteins were generated using the Human Protein Atlas (HPA) and UniProt databases.

### Protein interaction and functional analyses

The STRING (Search Tool for the Retrieval of Interacting Genes/Proteins) database was searched for all secreted proteins and human nasal epithelial membrane proteins. And the interaction between secreted proteins and human nasal epithelial membrane proteins was generated with default settings. Then the KEGG Orthology-Based Annotation System (KOBAS) [73] with default settings was utilized for functional enrichment analysis of all proteins.

### Statistical processing

Significant intergroup differences were assessed with the two-tailed Student *t*-test. Chi-square or Fisher’s exact test was utilized for significance analysis of Gene Ontology (GO) enrichment according to data features and hypergeometric test of KEGG.

## Results

### Genomic assembly and annotation

**Fig. 1:**
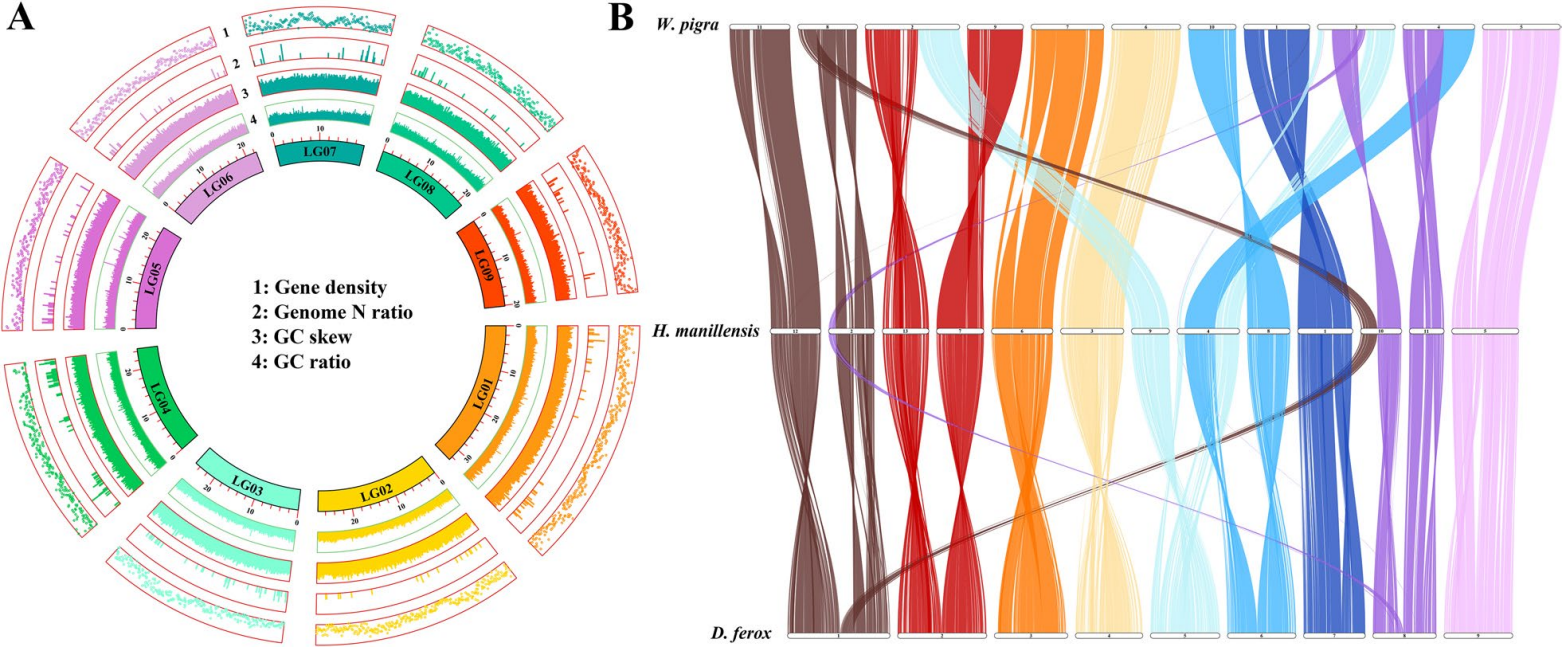
Statistical and collinearity analysis of *D. ferox* genomic assembly information. **A** Statistical circle plot of genomic assembly information. Outer circle to inner circle represent gene density, genome N ratio, genome GC skew, and genome GC ratio. **B** Genomic collinearity analysis of *D. ferox*, *Hirudinaria manillensis*, and *Whitmania pigra*. Only collinearity information between chromosomes was presented, and collinearity of contigs was omitted

Firstly, the cytochrome *c* oxidase subunit 1 (*COI*) gene sequence of the target species in this study was obtained through Sanger sequencing. By phylogenetically comparing it with the *COI* sequences of *D. ferox* and other species in NCBI, the target species was confirmed to be *D. ferox* (Additional file 1: Figure S1). Genomic DNA was harvested from mature *D. ferox* individuals, and then short/long-read sequencing strategies were implemented for hybrid sequencing. PacBio third-generation sequencing generated a total of 27.09 Gb (118.65×) of clean data, with an average read length of 10.63 Kb (Additional file 2: Table S1), while Illumina sequencing read coverage was 192×. Contaminants were eliminated after combining, cleaning, and correcting sequence reads. The final assembled genomic size was 228.33 Mb with a contig N50 of 2.16 Mb, which was consistent with the k-mer estimation. Hi-C assembly was utilized for mounting the scaffold of *D. ferox* on nine chromosomes, and the mounting rates were all > 96% (Fig. 1A). Over 98% of short reads could be matched to the reference genome (Additional file 2: Table S2). By evaluating 303 conserved core genes, BUSCO revealed that genomic integrity surpassed 95%, while CEGMA indicated that genomic integrity was > 97% in eukaryotes (Additional file 2: Table S3), indicating excellent quality of genomic assembly. Furthermore, we conducted statistical analysis on the GC (guanine/cytosine) ratio, GC skew, unknown bases (N), and gene density of each chromosome on the genome, and the findings suggested certain discrepancies across chromosomes (Fig. 1A). According to the prediction, the assembled genome comprised 19,242 protein-coding genes (Additional file 2: Table S4). Furthermore, eight miRNAs, 133 rRNAs, 1308 tRNAs, and 221 pseudogenes were predicted to be detected (Additional file 2: Table S5). Further functional annotation of all genes revealed that 16,525/19,242 genes had distinct functions in at least one database (Additional file 2: Table S6).

**Fig. 2:**
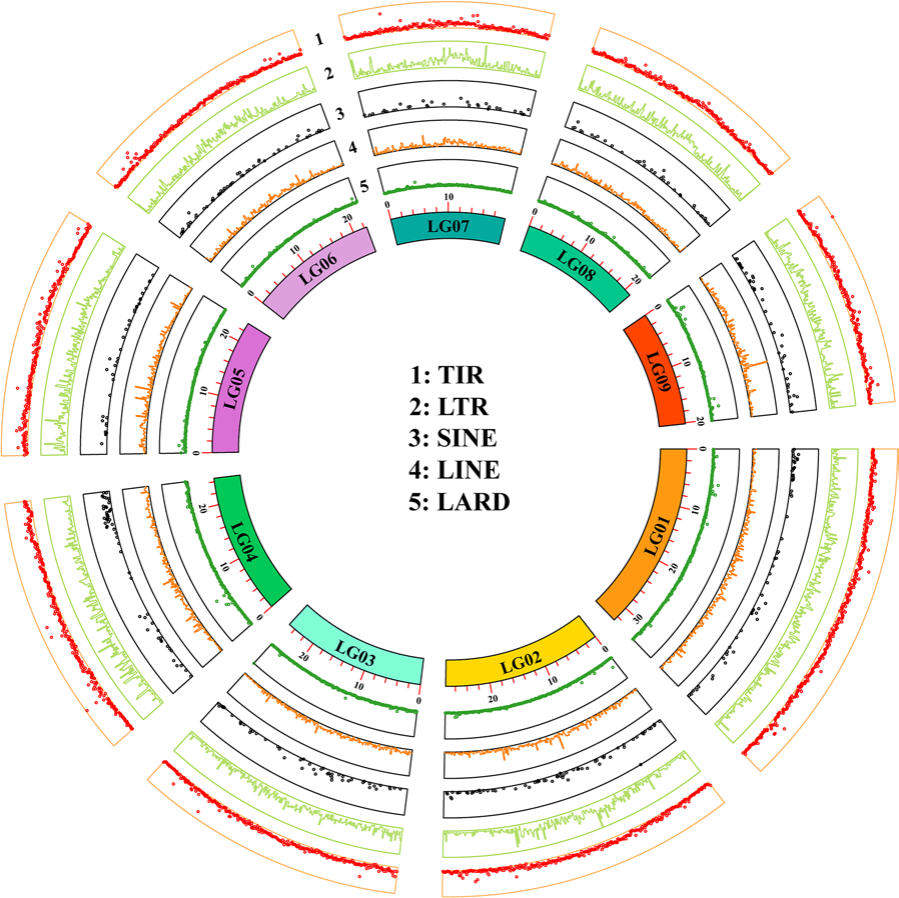
Distribution of repeat sequences in *D. ferox* genome

Furthermore, a genome-wide repeat sequence database was established with structural and de novo prediction techniques. The repeat sequence station in *D. ferox* accounted for 21.68% of the whole genome (Additional file 2: Table S7), with a total of 210,447 repeat sequences. The three most common forms of repeats in the genome were large retrotransposon derivatives (LARD), long interspersed nuclear elements (LINE), and long terminal repeats (LTR) (Additional file 2: Table S6). The genomic distribution of large repeats showed repetitions on several chromosomes and chromosomal regions, and discrepancies existed in sequence distribution (Fig. 2). We also compared the genomes of *D. ferox* and *H. manillensis* (unpublished data from our laboratory), as well as *W. pigra* (unpublished data from our laboratory). The collinearity in the genome was examined (Fig. 1B). The collinearity analysis reveals a high degree of chromosomal synteny between D. ferox, H. manillensis, and W. pigra. Notably, there is substantial homology between the p arm of chromosome 12 in H. manillensis and the p arm of chromosome 1 in D. ferox, and also between the q arm of chromosome 2 in H. man and the q arm of chromosome 1 in D. ferox. Additionally, a high degree of homology is also found between the p arm of chromosome 10 in H. manillensis and the p arm of chromosome 8 in D. ferox, and between the whole of chromosome 11 in H. manillensis and chromosome 8 in D. ferox. At the same time, several events of chromosomal inversion/translocation existed between *D. ferox* and *H. manillensis*, *W. pigra*, indicating a high degree of evolutionary resemblance.

### Phylogenetics and rapidly evolving genes of *D. ferox*

**Fig. 3:**
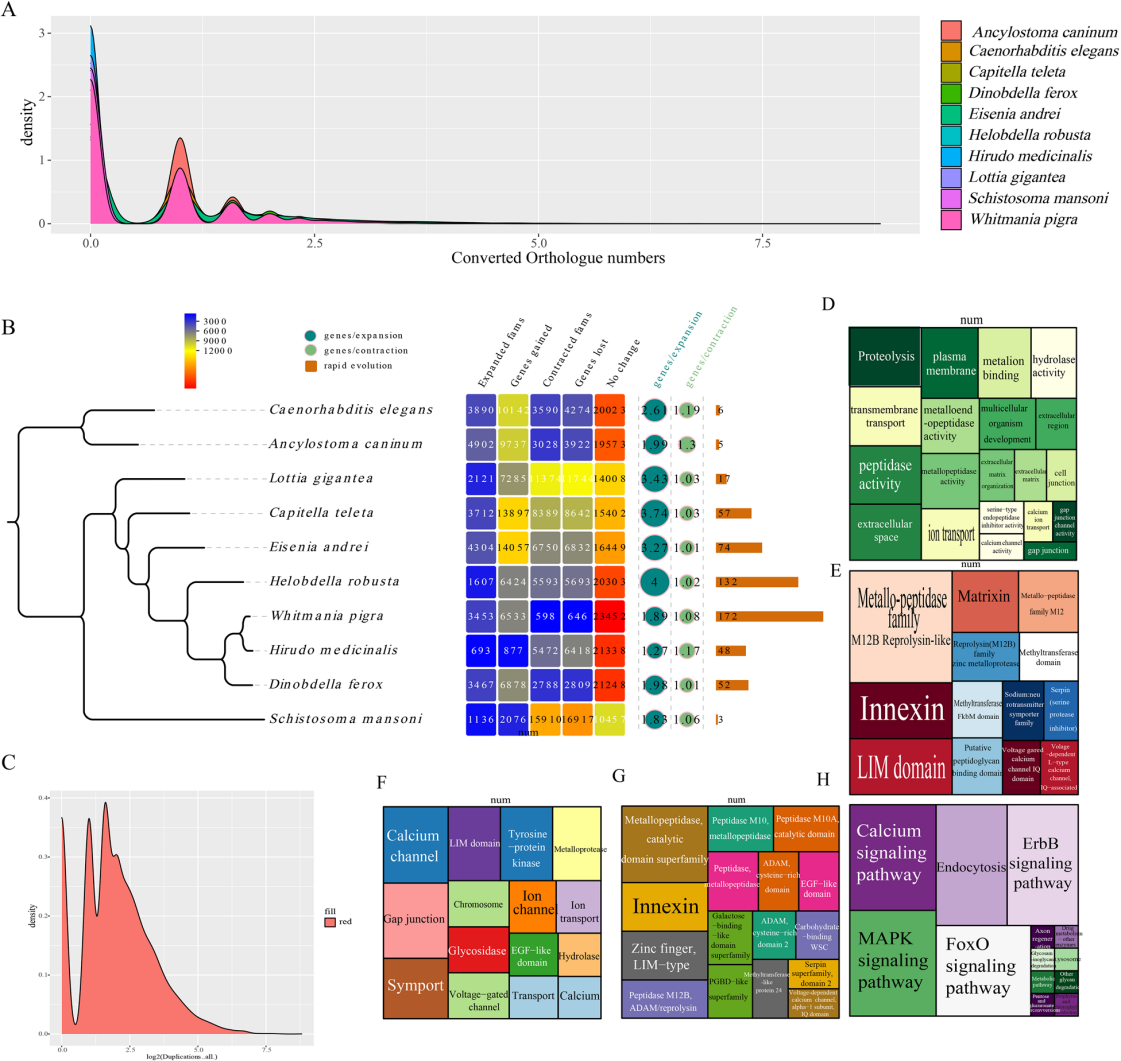
Phylogenetic and rapidly evolving genes in *D. ferox*. **A** statistical density plot of homologous genes in *D. ferox* and nine other species; **B** phylogenetic map of *D. ferox* and nine other species. Heatmap presents the number of genes expanding and contracting in all species, while the bar chart displays rapidly evolving genes in all species; **C** density map of gene repeat distribution. **D**–**H** GO enrichment analysis, InterPro enrichment analysis, KEGG enrichment analysis, KeyWords enrichment analysis, and Pfam domain annotation of rapidly evolving genes

The phylogeny of *D. ferox* was reconstructed by combining the de novo assembled *D. ferox* genome from this study with nine previously published genomes from *Caenorhabditis elegans*, *Ancylostoma caninum*, *Lottia gigantea*, *Capitella teleta*, *Eisenia andrei*, *Helobdella robusta*, *W. pigra*, *Hirudo medicinalis*, and *Schistosoma mansoni*. A total of 134 single-copy orthologous genes were identified across 10 separate species. Great variations across species existed in copies of gene families (Fig. 3A). The findings of our study indicated that *D. ferox, W. pigra*, and *H. medicinalis* belonged to the same clade (Fig. 3B). It agreed with the findings of morphological taxonomy. For exploring the changes in gene families, the shrinking and growing of gene families were examined in 10 different species. A total of 3467 genes were enlarged, 2788 genes contracted, and 2809 genes became missing in the *D. ferox* population (Fig. 3B, Additional file 2: Table S8). Fifty-two of these gene families revealed a rapid evolution (Fig. 3B). The duplication of genes in the genome was analyzed and the results indicated that various genes in the *D. ferox* genome displayed distinct variations in copy number (Fig. 3C). Functional analysis of rapidly evolving genes revealed that the functions of these genes were correlated with proteolysis, transmembrane transport, metalloendopeptidase activity, peptidase activity, extracellular space, matrixin, putative peptidoglycan binding domain, gap junction, and glycosidase. It was worth noting that a strong connection existed between processes and biological functions (Fig. 3D–H, Additional file 2: Tables S9–13). Thus, rapid evolution and expansion of these genes assisted in adapting to the adhesion environment of mammalian nasal parasites. These rapidly evolving genes included many genes closely correlated with proteolysis, extracellular matrix (ECM) degradation, and extracellular junctions.

### Interaction with human nasal cavity

**Fig. 4:**
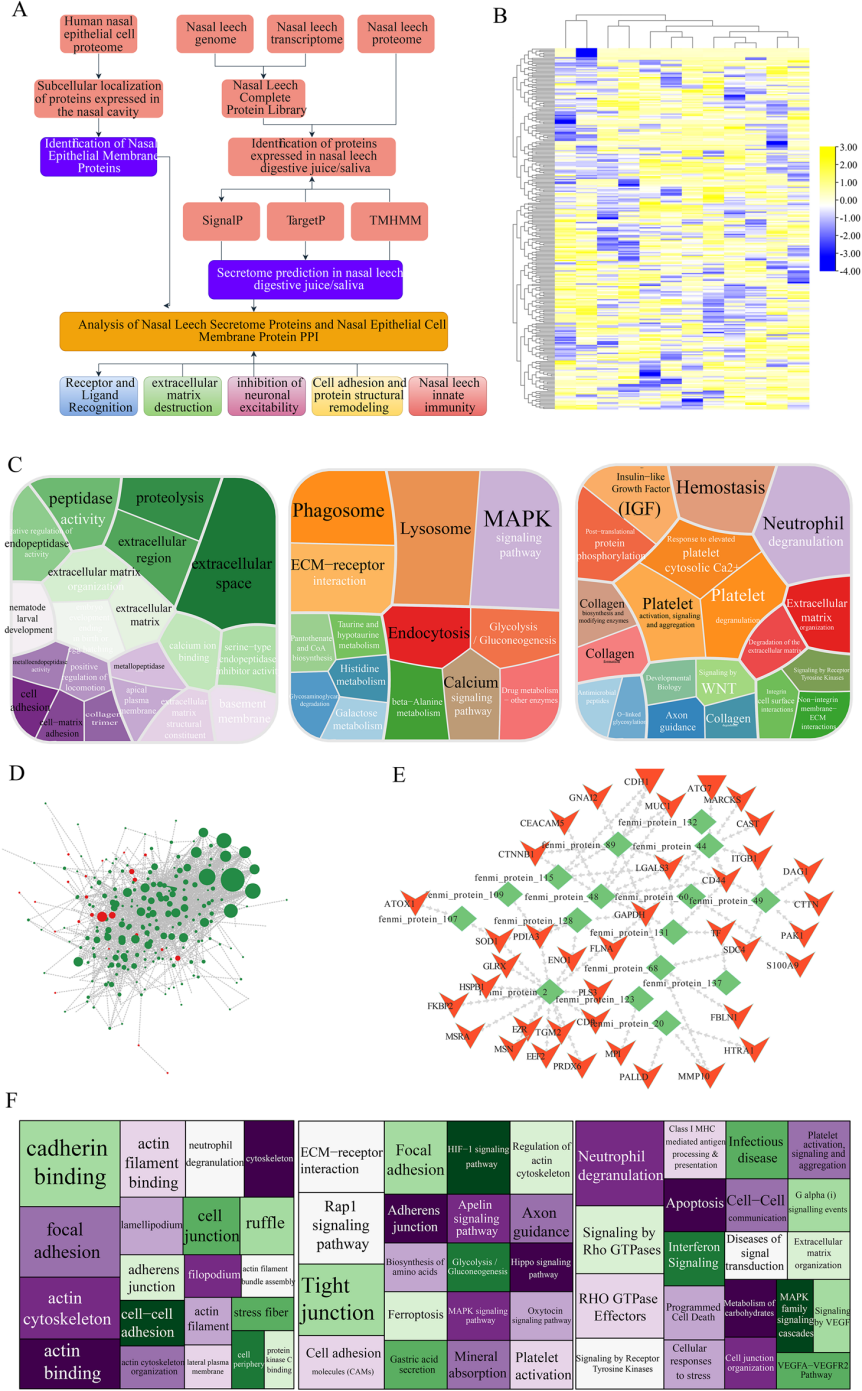
Interaction analyses of secreted proteins and nasal epithelial membrane proteins. **A** A pipeline of secreted protein prediction and interaction analysis process between secreted proteins of *D. ferox* and nasal epithelial membrane proteins. This study integrated the data for human nasal epithelial cell proteome, *D. ferox* genome, *D. ferox* transcriptome, and *D. ferox* proteome. **B** Expression profile of membrane proteins in nasal epithelial cells. **C** Functional enrichment analysis of membrane proteins in nasal epithelial cells. **D** Interaction network between nasal epithelial membrane proteins and *D. ferox* secreted proteins. **E** Core interaction network between nasal epithelial membrane proteins and *D. ferox* secreted proteins. **F** Functional analysis of genes in the interaction network

The analytical workflow was implemented for examining the potential interacting mechanism between *D. ferox* and mammalian nasal epithelium (Fig. 4A). RNA-seq data acquired from the digestive tract and saliva of *D. ferox* species were then aligned. For generating new transcripts non-aligned to the genome, a de novo assembly approach was adopted. And a comprehensive database of proteins was successfully established. Through proteomics analysis, a total of 446 proteins were detected. A further attempt was made to anticipate the level of secretion for each protein. A total of 187 proteins were predicted using SignalP as secreted proteins (Additional file 2: Table S14), 194 proteins as projected using TargetP as secreted proteins (Additional file 2: Table S15), and TMHMM predicted a total of 85 proteins as secreted proteins (Additional file 2: Table S16). Similarly, all findings were merged, and the secreted proteins of *D. ferox* identified. The expression patterns of 2198 proteins were collected from the proteome of human nasal epithelial cells from public sources. These proteins were predominantly expressed in nasal epithelial cells. This was consistent with the results of subcellular localization prediction, denoting that 263 proteins belonged to epithelial membrane proteins (Fig. 4B). For functional examination, these secreted proteins were correlated with peptidase activity, proteolysis, ECM, ECM-receptor interaction, hemostasis, collagen production, and neutrophil degranulation (Fig. 4C). Then, protein–protein interaction (PPI) analysis was performed on all released proteins as well as proteins of the nasal epithelial membrane (Fig. 4D, E). CD44, CDH1, LGALS3, MUC1, CTNNB1, PDIA3, and other proteins on the surface of the nasal epithelial cell membrane might be the most important cell membrane surface proteins (Fig. 4E). MUC4, MUC5AC, CTSB, SERPINB3, SERPINB8, LAMA3, and P4HB were the most important ones. The functional study of all proteins in the interaction network hinted at their roles in cadherin binding, cell–cell adhesion, focal adhesion, tight junction, cell–cell communication, neutrophil degranulation, and the Rap1 signaling pathway (Fig. 4F). These functional pathways were further categorized into the following five distinct modules of receptor/ligand recognition, destruction of ECM, inhibition of neuronal excitability, cell adhesion and protein structural remodeling, and nasal leech innate immunity. It was assumed that parasitism of *D. ferox* may be mediated by the changes in biological processes and pathways (Fig. 4A).

### Model of interactions between secretome and nasal mucosa of mammals

**Fig. 5:**
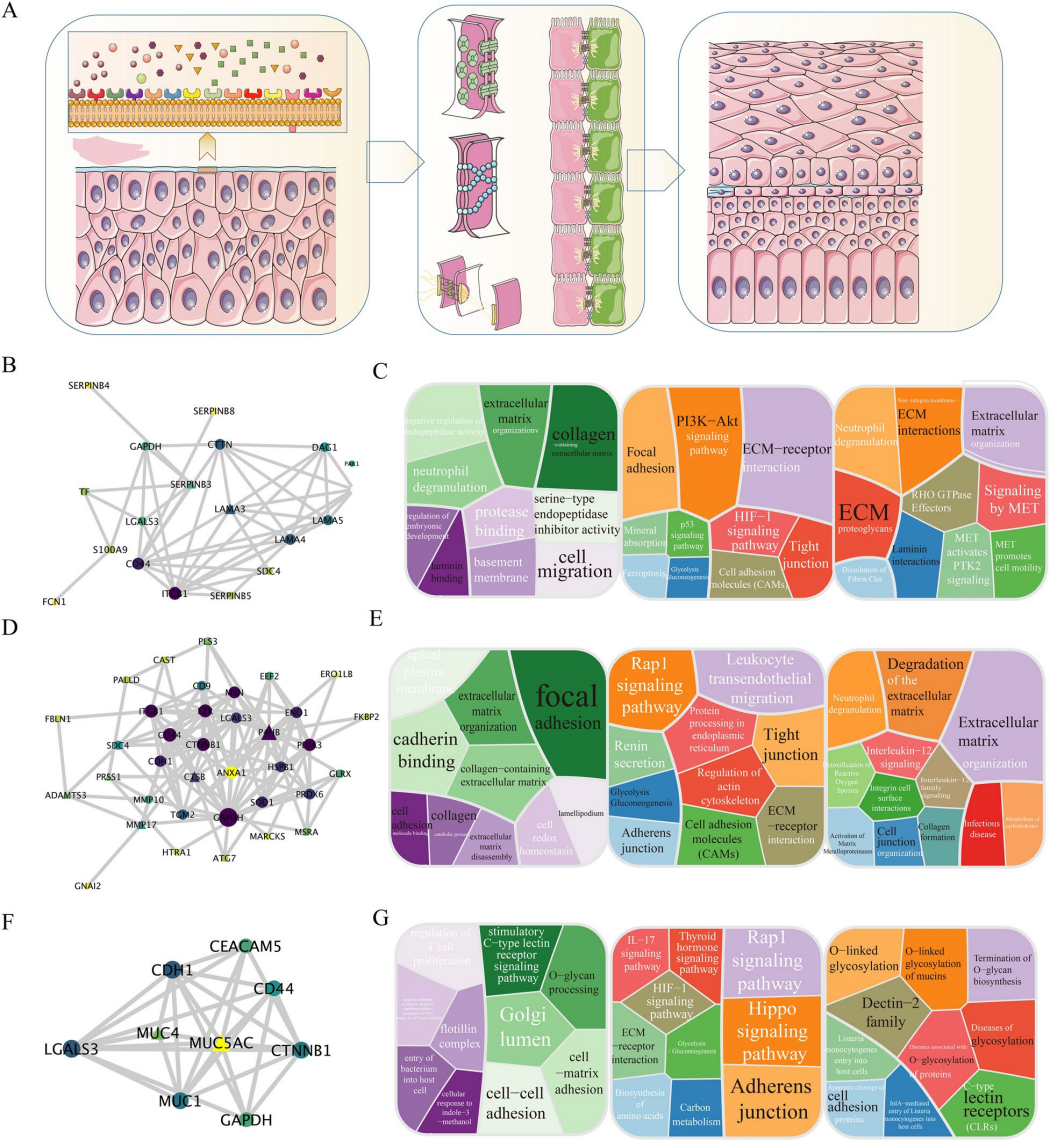
Molecular model of nasal cavity of *D. ferox* parasitic mammals. **A** Schematic diagram of cell junctions in nasal cavity of *D. ferox* parasitic mammals. When nasal cell membrane proteins are stimulated by *D. ferox*-secreted proteins, receptors on the cell membrane surface may be activated, causing changes in signaling pathways in nasal epithelial cells, thereby enabling them to initiate adhesion-related pathways. Finally, tight cellular junctions form between nasal epithelial cells and *D. ferox* epidermal cells. **B** Interaction network of pattern recognition-related genes. **C** Pattern recognition-related gene function analysis. **D** Interaction network of genes involved in ECM destruction and reconstruction. **E** Functional analysis of genes correlated with destruction and reconstruction of ECM. **F** Interaction network of mucin-related genes. **G** Functional analysis of mucin-related genes

**Fig. 6:**
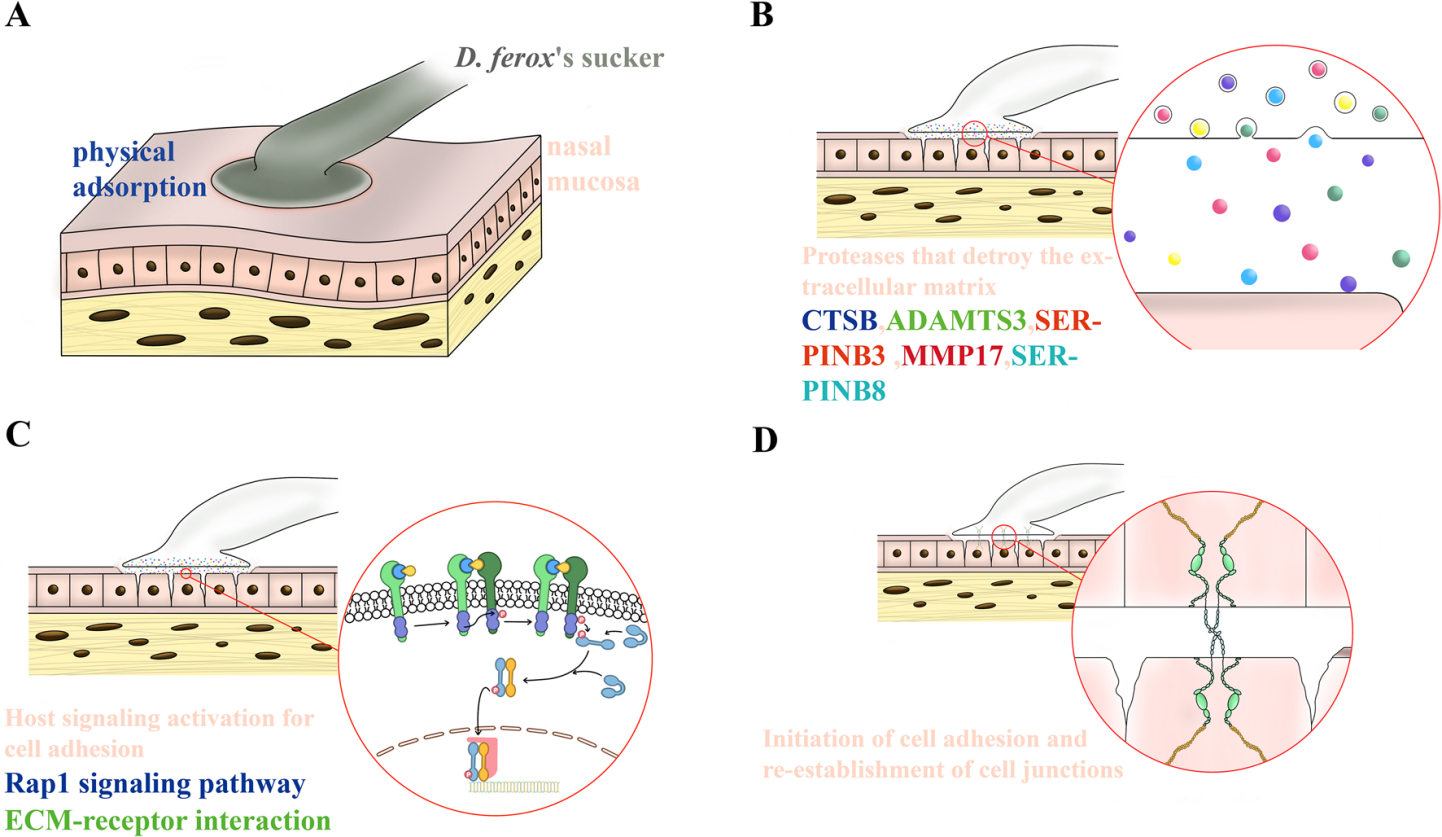
Mechanism and models of *D. ferox*-specific parasite mammalian nasal mucosa derived from multi-omics analysis and proposed in our research. **A** Suction cups will be used to adsorb *D. ferox* onto the mucosal tissue, and physical activity will complete the initial attachment; **B** *D. ferox* secretes a number of proteases that degrade the extracellular matrix and connections of the host mucosa; **C** *D. ferox* secretes ligands that trigger adhesion-related signaling pathways by binding to receptors on mucosal epithelial cells. **D** Reconstruction of the extracellular matrix and cell connections between mucosal epithelial cells and body surface cells of *D. ferox*

Based upon the findings of the prior analysis, the interaction network between *D. ferox* and nasal epithelium had five different functional modules. These modules included receptor and ligand recognition, inhibition of neuronal excitability, destruction of ECM, cell adhesion and protein structural remodeling, and nasal leech innate immunity (Figs. 5A, 6). Thus it was assumed that there is not only adhesion between ECM proteins between *D. ferox* body surface cells and mammalian nasal epithelial cells, but also reinforced adhesion with a more stable physical structure. Both types of cells adhered to ECM proteins (Fig. 5A). This adhesion offered a stable habitat for parasitism in the nasal canal, and it will not slip off regardless of how frequently animals breathe or how violently they sneeze. Based upon prior functional analyses, the interaction network might be divided into four distinct subnetworks, including PPI networks related to pattern recognition of the nasal leech in the nasal cavity (Fig. 5B), ECM protein degradation and rebuilding (Fig. 5D), and mucin (Fig. 5F) and P4HB-mediated interactions (Additional file 1: Figure S2). Pattern recognition-related proteins, for example, are intimately connected to a variety of pathways, including the PI3K-Akt signaling pathway, interaction between ECM and receptors, cell motility, laminin interactions, and others (Fig. 5C). Degradation of ECM, tight junctions, focal adhesions, and other processes are all associated with proteins involved in the breakdown and repair of ECM (Fig. 5E). The mucin-related network is correlated with several biological processes, such as processing of o-glycans, cell–cell adhesion, and O-linked glycosylation (Fig. 5G). Furthermore, the PPI network regulated by P4HB is closely correlated with membrane docking, extracellular exosomes, and glycolysis/gluconeogenesis, etc.

## Discussion

Aided by extensive research methodologies, a high-quality genome offers a valuable resource for modeling the parasitic features of *D. ferox*. Fifty-two rapidly evolving gene families were detected by comparative genomics. According to functional analyses, these gene families were closely correlated with proteolysis, transmembrane transport, metalloendopeptidase activity, peptidase activity, extracellular space, matrixin, putative peptidoglycan binding domain, gap junction, and glycosidase. Proteolysis [7], metalloendopeptidase activity [37], peptidase [76], and peptidoglycan-binding domain were all associated with the hydrolysis of peptide bonds, and glycosidase mediated protein glycosylation [27, 33, 44]. This implied that these rapidly evolving genes were probably involved in the destruction and remodeling of ECM. When *D. ferox* juvenile parasitizes the nasal mucosa of mammals, metalloendopeptidase, peptidase [67], and other peptide bond hydrolases are initially released for breaching the barriers of ECM and cell connections on nasal mucosa, causing nasal hemorrhage and even animal mortality. This process is accompanied by the secretion and alteration of various glycoproteins (mucin).

In the interaction network between *D. ferox*-secreted proteins and human nasal epithelial membrane proteins, LAMA3 might act as a pattern recognition protein in *D. ferox* to mediate its attachment to the nasal cavity. Laminin has been known to mediate the attachment, migration, and organization of cells into tissues during embryonic development. After cell binding via a high-affinity receptor, LAMA3 interacted with other ECM components [10, 45, 60, 70]. As a complex glycoprotein, laminin is composed of three distinct polypeptide chains (alpha, beta, and gamma) interlinked by disulfide bonds. A cross-shaped molecule is composed of one long arm and three short arms with globules at each end. Alpha-3 is a subunit of laminin-5 (laminin-332 or epiligrin/kalinin/nicein), laminin-6 (laminin-311/K-laminin), and laminin-7 (laminin-321/KS-laminin) [3, 14, 29, 69]. LAMA3 functions as a membrane protein in nasal epithelial cells of CD44, CTTN, DAG1, ITGB1, PAK1, SDC4, and other receptor protein ligands. After receptor binding, it may facilitate ECM connection and attachment of body surface epithelial cells and nasal cavity epithelial cells [19, 23, 25, 26].

Regarding ECM destruction and regeneration, CTSB, ADAMTS3, SERPINB3, and SERPINB8 may play critical roles in *D. ferox* adhesion. SERPINB8 was found to be vital for cell–cell adhesion mediated by epithelial desmosomes [58]. Likely involved in intracellular protein degradation and turnover, CTSB cleaved extracellular stromal phosphoglycoprotein MEPE [4, 9, 41]. As a matrix metalloproteinase, MMP17 may destroy ECM components such as fibrin [12, 64, 72]. Additionally, ADAMTS3 cleaved type II collagen propeptides prior to fibril assembly [20, 35]. This implies that *D. ferox* juveniles release a significant quantity of matrix proteases prior to parasitizing the nasal mucosa of mammals. The *D. ferox* juvenile would degrade ECM of nasal mucosa through cell matrix-destroying enzymes of CTSB, ADAMTS3, SERPINB3, SERPINB8, and MMP17, hence facilitating the formation of cellular connections between epithelial cells of the nasal cavity. Furthermore, mucins (MUC5AC and MUC4) served as crucial attachment proteins in the early stages of attachment [28, 65, 75]. MUC5AC and MUC4 probably interacted with MUC1, CDH1, and LGALS3 on the membrane of nasal epithelial cells. During the early stage of parasitism, *D. ferox* adheres to nasal mucosa through interactions.

Furthermore, a P4HB-mediated sub-network operated inside the interaction network. Secreted protein might interact with 16 nasal epithelial membrane proteins. P4HB catalyzed the formation, breakage, and rearrangement of disulfide bonds [5, 40, 43, 77]. It appeared to act as a reductase at the cell surface, cleaving disulfide bonds of proteins bound to cells. It enabled structural changes in outer surface proteins. P4HB enhanced protein aggregation at a low dose and cooperated with other molecular chaperones in the structural alteration of thyroglobulin (TG) precursors during hormone biosynthesis. It was hypothesized that P4HB promoted the re-formation of disulfide linkages between nasal epithelial proteins and body surface epithelial membrane proteins or secreted proteins. The formation of disulfide bonds significantly enhanced the adhesion between *D. ferox* and the nasal cavity, creating an extremely stable adhesion environment for the nasal parasite life of *D. ferox* from juvenile to adult. Individual cells in diverse surroundings attach to ECM and their neighbors through the integrin and cadherin complexes. These dynamic interactions secure the creation and maintenance of complex tissues. A growing body of evidence has highlighted the roles of Rap1 GTPase and its accompanying signaling network [30, 49]. Also, the Rap1 signaling pathway plays an important role in the adhesion interacting network. Secreted protein ligands might bind to G protein-coupled receptors in nasal epithelial cells and activate the intracellular Rap1 signaling pathway. Cell adhesion between nasal epithelium and epidermal cells could be induced via an active Rap1 signaling pathway.

Approximately one third of the two million eukaryotes are parasites, and all organisms act as hosts or parasites. Parasitism between the parasite and its host determines their respective selection processes and life histories. Currently, numerous prevalent mammalian parasites complete their physical attachment to certain host areas through specialized organs and specific body parts. Lice feet are specialized as hair-hooking grippers to assist them in parasitizing on mammalian hair, while the scolex of tapeworms has evolved suckers, rostellum, and tiny hooks to aid in clinging to the mucosa of the small intestine of humans. Most blood-sucking leech species rely on suckers to stick to the host surface and detach after a brief blood-sucking session. *Dinobdella ferox* is parasitic on the nasal mucosa of mammals from its juvenile stage and completes its maturation through a lengthy parasitic cycle of several months. During parasitism, *D. ferox* withstands intense sneezing and breathing by the host due to foreign body sensation. However, its adherence stability and spatial structure are vastly superior to that of other leeches by interspecific molecular interactions (Fig. 6). Besides the secretion of mucins, chemical adhesion may be partially mediated through its suckers. Also, the host mucosa is compromised through the release of various proteases, activating the host signaling system to commence the reconstruction of ECM and simultaneously mediate the development of new chemical bonds between proteins. The tight intercellular connection enables a particularly stable and long-term parasite on the nasal epithelium of its host, providing a local habitat for completing its life cycle. The effectiveness of the parasitic lifestyle is never challenged throughout its evolutionary history. The host provides not only food and shelter for any exploitive species, but also an effective mode of transmission. Our paradigm of parasitism is probably found not only in *D. ferox* but also in numerous long-term adherent parasites in nature.

## Conclusions

Most mammalian parasites complete their physical attachment to certain host areas through specialized organs and specific body parts. The present study represents the first-ever attempt to propose a molecular model for specific parasitism. *Dinobdella ferox* and mammalian nasal epithelial cells formed a chemical adhesion with three major steps of pattern recognition, mucin connection, and breakdown and repair of ECM. This molecular model may serve as a practical reference for parasitism models of other species and a theoretical foundation for a molecular process of parasitism.

### Supplementary Information

**Additional file 1: Figure S1. **Molecular phylogenetic analysis of leech species by maximum likelihood method based on *COI* genes. The label with the red dot corresponds to the target leech species in this study. **Figure S2.** P4HB-mediated protein and protein interaction network. Data come from the STRING database

**Additional file 2:** **Table S1.** Data volume statistics of long-read sequencing in *D. ferox* genome. **Table S2.** Alignment rate statistics of next-generation sequencing data compared to the *D. ferox* genome. **Table S3.** The results of the integrity test of the *D. ferox* genome by BUSCO. **Table S4.** Statistics and annotation of genes and gene structure in *D. ferox* genome. **Table S5.** Identification of non-coding RNA genes in *D. ferox* genome. **Table S6.** Annotation statistics of protein-coding genes in *D. ferox* genome. **Table S7.** Identification and statistics of repeated sequences in *D. ferox* genome. **Table S8.** Gene family contraction and expansion analysis results. **Table S9. **KEGG enrichment analysis of rapidly evolving genes in *D. ferox* genome. **Table S10. **GO enrichment analysis of rapidly evolving genes in *D. ferox* genome. **Table S11.** InterPro (IPR) enrichment analysis of rapidly evolving genes in *D. ferox* genome. **Table S12. **KeyWords enrichment analysis of rapidly evolving genes in *D. ferox* genome. **Table S13. **Pfam domain enrichment analysis of rapidly evolving genes in *D. ferox* genome. **Table S14. **Prediction of the secretion ability of salivary protein in *D. ferox* genome by SignalP. **Table S15. **Prediction of the secretion ability of salivary protein in *D. ferox* genome by TargetP. **Table S16. **Prediction of the secretion ability of salivary protein in *D. ferox* genome by TMHMM.

## Data Availability

All of the raw data genearted in this study have been deposited in the National Center for Biotechnology Information (NCBI) under SRA accession PRJNA1005568 that is publicly accessible at https://www.ncbi.nlm.nih.gov/sra/PRJNA1005568. All other data are available from the authors upon reasonable request.

## Abbreviations

BUSCO: Benchmarking Universal Single-Copy Orthologs
CEGMA: Core Eukaryotic Genes Mapping Approach
ECM: Extracellular matrix
GO: Gene Ontology
GPS: Global Positioning System
HCD: Higher-energy collisional dissociation
HPA: Human Protein Atlas
KEGG: Kyoto Encyclopedia of Genes and Genomes
LARD: Large retrotransposon derivative
LINE: Long interspersed nuclear element
LTR: Long terminal repeat
NGS: Next-generation sequencing
PPI: Protein–protein interaction
