# Supplementary material for: Chromosome-level Dinobdella ferox genome provided a molecular model for its specific parasitism
Source: Parasit Vectors. 2023 Sep 11;16:322. doi: 10.1186/s13071-023-05837-7 (PMC10494388; doi:10.1186/s13071-023-05837-7)
Supplement: Supplementary file 2 — Additional file 2: Table S1. Data volume statistics of long-read sequencing in D. ferox genome. Table S2. Alignment rate statistics of next-generation sequencing data compared to the D. ferox genome. Table S3. The results of the integrity test of the D. ferox genome by BUSCO. Table S4. Statistics and annotation of genes and gene structure in D. ferox genome. Table S5. Identification of non-coding RNA genes in D. ferox genome. Table S6. Annotation statistics of protein-coding genes in D. ferox genome. Table S7. Identification and statistics of repeated sequences in D. ferox genome. Table S8. Gene family contraction and expansion analysis results. Table S9. KEGG enrichment analysis of rapidly evolving genes in D. ferox genome. Table S10. GO enrichment analysis of rapidly evolving genes in D. ferox genome. Table S11. InterPro (IPR) enrichment analysis of rapidly evolving genes in D. ferox genome. Table S12. KeyWords enrichment analysis of rapidly evolving genes in D. ferox genome. Table S13. Pfam domain enrichment analysis of rapidly evolving genes in D. ferox genome. Table S14. Prediction of the secretion ability of salivary protein in D. ferox genome by SignalP. Table S15. Prediction of the secretion ability of salivary protein in D. ferox genome by TargetP. Table S16. Prediction of the secretion ability of salivary protein in D. ferox genome by TMHMM. [file 13071_2023_5837_MOESM2_ESM.docx]

**Table S1** Data volume statistics of long-read sequencing in *D. ferox* genome.

| **Data Type** | **Reads num** | **Reads base** | **Reads LenN50** | **Reads LenMean** | **Reads LenMax** |
| --- | --- | --- | --- | --- | --- |
| Subreads | 2,548,108 | 27,091,272,968 | 12,976 | 10,632 | 118,459 |
| ZMWreads | 1,149,434 | 12,641,483,815 | 14,199 | 10,998 | 118,459 |

**Table S2** Alignment rate statistics of next-generation sequencing data compared to the *D. ferox* genome.

| **Species** | **Total reads** | **Mapped reads** | **Mapped(%)** | **Properly mapped reads** | **Properly mapped(%)** |
| --- | --- | --- | --- | --- | --- |
| *D. ferox* | 123,753,229 | 121,434,580 | 98.13 | 119,305,811 | 96.41 |

**Table S3** The results of the integrity test of the *D. ferox* genome by BUSCO.

| **Complete BUSCOs(C)** | **Complete and single-copy BUSCOs(S)** | **Complete and duplicated BUSCOs(D)** | **Fragmented BUSCOs(F)** | **Missing BUSCOs(M)** | **Total lineage BUSCOs** |
| --- | --- | --- | --- | --- | --- |
| 289 (95.38%) | 262 (86.47%) | 27 (8.91%) | 2 (0.66%) | 12 (3.96%) | 303 |

**Table S4** Statistics and annotation of genes and gene structure in *D. ferox* genome.

| **Item** | **Number/Length** |
| --- | --- |
| GeneNum | 19,242 |
| AveGenLen | 4,061.46 |
| ExonLen | 29,154,282 |
| AveExonLen | 1,515.14 |
| ExonNum | 131,763 |
| AveExonNum | 6.85 |
| CDSLen | 29,154,282 |
| AveCDSLen | 1,515.14 |
| CDSNum | 131,763 |
| AveCDSNum | 6.85 |
| IntronLen | 48,996,276 |
| AveIntronLen | 2,546.32 |
| IntronNum | 112,521 |
| AveIntronNum | 5.85 |

**Table S5** Identification of non-coding RNA genes in *D. ferox* genome.

| **RNA classification** | **Number** | **Family** |
| --- | --- | --- |
| miRNA | 8 | 6 |
| rRNA | 133 | 4 |
| tRNA | 1,308 | 25 |

**Table S6** Annotation statistics of protein-coding genes in *D. ferox* genome.

| **Annotation database** | **Annotated number** | **Percentage (%)** |
| --- | --- | --- |
| GO Annotation | 5,924 | 30.79 |
| KEGG Annotation | 8,472 | 44.03 |
| KOG Annotation | 12,192 | 63.36 |
| TrEMBL Annotation | 16,421 | 85.34 |
| Nr Annotation | 16,432 | 85.4 |
| All Annotated | 16,525 | 85.88 |

**Table S7** Identification and statistics of repeated sequences in *D. ferox* genome.

| **Type** | **Number** | **Length** | **Rate (%)** |
| --- | --- | --- | --- |
| ClassI | 210,447 | 49,505,801 | 21.68 |
| ClassI/DIRS | 2,065 | 553,186 | 0.24 |
| ClassI/LARD | 71,706 | 13,679,119 | 5.99 |
| ClassI/LINE | 51,555 | 15,330,678 | 6.71 |
| ClassI/LTR/Copia | 9,199 | 1,986,610 | 0.87 |
| ClassI/LTR/Gypsy | 64,328 | 19,171,301 | 8.4 |
| ClassI/LTR/Unknown | 7,980 | 2,116,015 | 0.93 |
| ClassI/PLE | 1,817 | 535,755 | 0.23 |
| ClassI/SINE | 506 | 97,883 | 0.04 |
| ClassI/TRIM | 772 | 578,752 | 0.25 |
| ClassI/Unknown | 519 | 43,442 | 0.02 |
| ClassII | 130,488 | 27,193,295 | 11.91 |
| ClassII/Crypton | 240 | 15,888 | 0.01 |
| ClassII/Helitron | 3,818 | 663,420 | 0.29 |
| ClassII/MITE | 1,037 | 170,404 | 0.07 |
| ClassII/Maverick | 5,379 | 869,652 | 0.38 |
| ClassII/TIR | 104,492 | 22,870,475 | 10.02 |
| ClassII/Unknown | 15,522 | 3,285,409 | 1.44 |
| PotentialHostGene | 3,770 | 1,133,371 | 0.5 |
| SSR | 1,926 | 255,318 | 0.11 |
| Unknown | 68,061 | 12,765,183 | 5.59 |
| Total | 414,692 | 79,648,092 | 34.88 |

**Table S8** Gene family contraction and expansion analysis results.

| **Species** | **Expanded fams** | **Genes gained** | **Genes/**  **expansion** | **Contracted fams** | **Genes lost** | **Genes/**  **contraction** | **No change** | **Avg. expansion** |
| --- | --- | --- | --- | --- | --- | --- | --- | --- |
| *S. mansoni* | 1136 (1) | 2076 | 1.83 | 15910 (2) | 16917 | 1.06 | 10457 | -0.539614 |
| *D. ferox* | 3467 (52) | 6878 | 1.98 | 2788 (0) | 2809 | 1.01 | 21248 | 0.147947 |
| *E. andrei* | 4304 (74) | 14057 | 3.27 | 6750 (0) | 6832 | 1.01 | 16449 | 0.262699 |
| *C. elegans* | 3890 (5) | 10142 | 2.61 | 3590 (1) | 4274 | 1.19 | 20023 | 0.213359 |
| *A. caninum* | 4902 (5) | 9737 | 1.99 | 3028 (0) | 3922 | 1.3 | 19573 | 0.211431 |
| *W. pigra* | 3453 (161) | 6533 | 1.89 | 598 (11) | 646 | 1.08 | 23452 | 0.214049 |
| *H. robusta* | 1607 (48) | 6424 | 4 | 5593 (0) | 5693 | 1.02 | 20303 | 0.0265789 |
| *H. medicinalis* | 693 (5) | 877 | 1.27 | 5472 (127) | 6418 | 1.17 | 21338 | -0.201469 |
| *C. teleta* | 3712 (57) | 13897 | 3.74 | 8389 (0) | 8642 | 1.03 | 15402 | 0.19107 |
| *L. gigantea* | 2121 (16) | 7285 | 3.43 | 11374 (1) | 11744 | 1.03 | 14008 | -0.162128 |

**Table S9** KEGG enrichment analysis of rapidly evolving genes in *D. ferox* genome*.*

| **Term** | **Database** | **ID** | **Input number** | **Background number** | **P-Value** | **Corrected P-Value** |
| --- | --- | --- | --- | --- | --- | --- |
| ErbB signaling pathway | KEGG PATHWAY | cel04012 | 16 | 38 | 6.38E-22 | 8.29E-21 |
| Calcium signaling pathway | KEGG PATHWAY | cel04020 | 17 | 62 | 1.42E-20 | 9.25E-20 |
| FoxO signaling pathway | KEGG PATHWAY | cel04068 | 16 | 59 | 2.26E-19 | 9.78E-19 |
| MAPK signaling pathway | KEGG PATHWAY | cel04010 | 17 | 79 | 4.97E-19 | 1.61E-18 |
| Endocytosis | KEGG PATHWAY | cel04144 | 16 | 105 | 7.00E-16 | 1.82E-15 |
| Glycosaminoglycan degradation | KEGG PATHWAY | cel00531 | 1 | 5 | 0.042614773 | 0.092332008 |
| Other glycan degradation | KEGG PATHWAY | cel00511 | 1 | 16 | 0.116094421 | 0.215603925 |
| Porphyrin and chlorophyll metabolism | KEGG PATHWAY | cel00860 | 1 | 21 | 0.147608578 | 0.222120432 |
| Pentose and glucuronate interconversions | KEGG PATHWAY | cel00040 | 1 | 22 | 0.153775683 | 0.222120432 |
| Drug metabolism - other enzymes | KEGG PATHWAY | cel00983 | 1 | 57 | 0.34375397 | 0.446880161 |
| Axon regeneration | KEGG PATHWAY | cel04361 | 1 | 86 | 0.468489156 | 0.553669003 |
| Lysosome | KEGG PATHWAY | cel04142 | 1 | 99 | 0.516438876 | 0.559475449 |
| Metabolic pathways | KEGG PATHWAY | cel01100 | 1 | 887 | 0.99850981 | 0.99850981 |

**Table S10** GO enrichment analysis of rapidly evolving genes in *D. ferox* genome.

| **Term** | **Database** | **ID** | **Input number** | **Background number** | **P-value** | **Corrected p-value** |
| --- | --- | --- | --- | --- | --- | --- |
| peptidase activity | Gene Ontology | GO:0008233 | 78 | 227 | 1.65E-98 | 3.28E-96 |
| proteolysis | Gene Ontology | GO:0006508 | 79 | 365 | 3.88E-86 | 3.84E-84 |
| metalloendopeptidase activity | Gene Ontology | GO:0004222 | 60 | 106 | 6.10E-86 | 4.02E-84 |
| extracellular space | Gene Ontology | GO:0005615 | 75 | 303 | 1.81E-85 | 8.98E-84 |
| metallopeptidase activity | Gene Ontology | GO:0008237 | 60 | 118 | 9.63E-84 | 3.81E-82 |
| transmembrane transport | Gene Ontology | GO:0055085 | 79 | 451 | 1.02E-79 | 3.35E-78 |
| plasma membrane | Gene Ontology | GO:0005886 | 75 | 875 | 1.01E-54 | 2.84E-53 |
| extracellular matrix organization | Gene Ontology | GO:0030198 | 34 | 40 | 3.02E-53 | 7.48E-52 |
| ion transport | Gene Ontology | GO:0006811 | 56 | 405 | 5.38E-51 | 1.18E-49 |
| extracellular matrix | Gene Ontology | GO:0031012 | 31 | 45 | 1.46E-46 | 2.89E-45 |
| multicellular organism development | Gene Ontology | GO:0007275 | 54 | 450 | 3.39E-46 | 6.11E-45 |
| membrane | Gene Ontology | GO:0016020 | 145 | 6222 | 3.20E-40 | 5.29E-39 |
| hydrolase activity | Gene Ontology | GO:0016787 | 60 | 831 | 1.39E-39 | 2.12E-38 |
| serine-type endopeptidase inhibitor activity | Gene Ontology | GO:0004867 | 27 | 51 | 2.57E-38 | 3.64E-37 |
| positive regulation of vulval development | Gene Ontology | GO:0040026 | 25 | 38 | 2.29E-37 | 3.02E-36 |
| metal ion binding | Gene Ontology | GO:0046872 | 70 | 1381 | 6.36E-37 | 7.87E-36 |
| integral component of membrane | Gene Ontology | GO:0016021 | 136 | 5882 | 8.13E-37 | 9.27E-36 |
| negative regulation of endopeptidase activity | Gene Ontology | GO:0010951 | 27 | 60 | 8.42E-37 | 9.27E-36 |
| calcium channel activity | Gene Ontology | GO:0005262 | 22 | 24 | 2.65E-35 | 2.76E-34 |
| voltage-gated ion channel activity | Gene Ontology | GO:0005244 | 22 | 25 | 4.95E-35 | 4.67E-34 |

**Table S11** IPR enrichment analysis of rapidly evolving genes in *D. ferox* genome.

| **Term ID** | | **Description** | **Observed gene count** | | **BG gene count** | **Strength** | **FDR** |
| --- | --- | --- | --- | --- | --- | --- | --- |
| IPR024079 | Metallopeptidase, catalytic domain superfamily | | | 22 | 94 | 1.62 | 2.92E-23 |
| IPR000990 | Innexin | | | 14 | 21 | 2.07 | 5.06E-19 |
| IPR001781 | Zinc finger, LIM-type | | | 14 | 57 | 1.64 | 2.95E-14 |
| IPR033739 | Peptidase M10A, catalytic domain | | | 10 | 23 | 1.88 | 1.41E-11 |
| IPR001590 | Peptidase M12B, ADAM/reprolysin | | | 11 | 41 | 1.67 | 3.53E-11 |
| IPR021190 | Peptidase M10A | | | 10 | 30 | 1.77 | 8.34E-11 |
| IPR001818 | Peptidase M10, metallopeptidase | | | 10 | 31 | 1.75 | 9.41E-11 |
| IPR006026 | Peptidase, metallopeptidase | | | 10 | 36 | 1.69 | 2.92E-10 |
| IPR036365 | PGBD-like superfamily | | | 8 | 15 | 1.97 | 1.24E-09 |
| IPR026913 | Methyltransferase-like protein 24 | | | 7 | 11 | 2.05 | 1.38E-08 |
| IPR006586 | ADAM, cysteine-rich domain | | | 8 | 27 | 1.72 | 4.59E-08 |
| IPR002477 | Peptidoglycan binding-like | | | 6 | 10 | 2.02 | 5.38E-07 |
| IPR002077 | Voltage-dependent calcium channel, alpha-1 subunit | | | 6 | 11 | 1.98 | 7.65E-07 |
| IPR041645 | ADAM cysteine-rich domain 2 | | | 7 | 26 | 1.68 | 1.23E-06 |
| IPR025714 | Methyltransferase domain | | | 6 | 16 | 1.82 | 3.90E-06 |
| IPR014873 | Voltage-dependent calcium channel, alpha-1 subunit, IQ domain | | | 5 | 6 | 2.17 | 4.37E-06 |
| IPR031649 | Voltage-dependent L-type calcium channel, IQ-associated domain | | | 5 | 8 | 2.04 | 1.14E-05 |
| IPR000175 | Sodium:neurotransmitter symporter | | | 6 | 23 | 1.66 | 2.01E-05 |
| IPR037272 | Sodium:neurotransmitter symporter superfamily | | | 6 | 23 | 1.66 | 2.01E-05 |
| IPR015557 | Leukocyte elastase inhibitor (serpin B1) | | | 5 | 13 | 1.83 | 6.28E-05 |

**Table S12** KeyWords enrichment analysis of rapidly evolving genes in *D. ferox* genome.

| **Term ID** | **Description** | **Count** | **BG gene count** | **Strength** | **False discovery rate** |
| --- | --- | --- | --- | --- | --- |
| KW-0303 | Gap junction | 14 | 21 | 2.07 | 3.19E-20 |
| KW-0440 | LIM domain | 14 | 56 | 1.64 | 7.51E-16 |
| KW-0813 | Transport | 26 | 591 | 0.89 | 2.22E-13 |
| KW-0407 | Ion channel | 19 | 299 | 1.05 | 3.85E-12 |
| KW-0406 | Ion transport | 20 | 356 | 1 | 5.65E-12 |
| KW-0482 | Metalloprotease | 10 | 53 | 1.52 | 3.05E-10 |
| KW-1133 | Transmembrane helix | 56 | 4292 | 0.36 | 2.47E-08 |
| KW-0107 | Calcium channel | 5 | 7 | 2.1 | 3.42E-07 |
| KW-0732 | Signal | 28 | 1427 | 0.54 | 6.71E-07 |
| KW-0769 | Symport | 6 | 23 | 1.66 | 8.76E-07 |
| KW-0378 | Hydrolase | 15 | 427 | 0.79 | 1.84E-06 |
| KW-0862 | Zinc | 25 | 1225 | 0.56 | 1.84E-06 |
| KW-0479 | Metal-binding | 25 | 1476 | 0.47 | 4.85E-05 |
| KW-0677 | Repeat | 14 | 489 | 0.7 | 4.98E-05 |
| KW-0851 | Voltage-gated channel | 5 | 57 | 1.19 | 0.0012 |
| KW-0106 | Calcium | 9 | 272 | 0.77 | 0.0014 |
| KW-0245 | EGF-like domain | 7 | 158 | 0.89 | 0.0018 |
| KW-0829 | Tyrosine-protein kinase | 3 | 12 | 1.64 | 0.0031 |
| KW-0371 | Homeobox | 8 | 243 | 0.76 | 0.0036 |
| KW-1015 | Disulfide bond | 12 | 580 | 0.56 | 0.0051 |
| KW-0238 | DNA-binding | 11 | 572 | 0.53 | 0.0171 |
| KW-0158 | Chromosome | 3 | 33 | 1.2 | 0.0375 |
| KW-0326 | Glycosidase | 3 | 34 | 1.19 | 0.0392 |

**Table S13** Pfam domain enrichment analysis of rapidly evolving genes in *D. ferox* genome.

| **Term ID** | **Description** | **Observed gene count** | **Background gene count** | **Strength** | **FDR** |
| --- | --- | --- | --- | --- | --- |
| PF00876 | Innexin | 14 | 21 | 2.07 | 4.82E-19 |
| PF00412 | LIM domain | 14 | 57 | 1.64 | 2.11E-14 |
| PF00413 | Matrixin | 10 | 31 | 1.75 | 7.94E-11 |
| PF13582 | Metallo-peptidase family M12B Reprolysin-like | 10 | 30 | 1.77 | 7.94E-11 |
| PF13688 | Metallo-peptidase family M12 | 9 | 25 | 1.8 | 5.73E-10 |
| PF13574 | Metallo-peptidase family M12B Reprolysin-like | 9 | 28 | 1.75 | 1.12E-09 |
| PF13583 | Metallo-peptidase family M12B Reprolysin-like | 9 | 28 | 1.75 | 1.12E-09 |
| PF13383 | Methyltransferase domain | 7 | 10 | 2.09 | 5.03E-09 |
| PF01471 | Putative peptidoglycan binding domain | 7 | 11 | 2.05 | 7.28E-09 |
| PF01421 | Reprolysin (M12B) family zinc metalloprotease | 8 | 33 | 1.63 | 9.48E-08 |
| PF05050 | Methyltransferase FkbM domain | 7 | 21 | 1.77 | 2.12E-07 |
| PF08763 | Voltage gated calcium channel IQ domain | 5 | 6 | 2.17 | 2.77E-06 |
| PF00209 | Sodium:neurotransmitter symporter family | 6 | 23 | 1.66 | 1.32E-05 |
| PF16905 | Voltage-dependent L-type calcium channel, IQ-associated | 5 | 11 | 1.9 | 2.20E-05 |
| PF00079 | Serpin (serine protease inhibitor) | 5 | 16 | 1.74 | 9.34E-05 |
| PF14843 | Growth factor receptor domain IV | 4 | 5 | 2.15 | 0.0001 |
| PF08516 | ADAM cysteine-rich | 5 | 19 | 1.67 | 0.00017 |
| PF00757 | Furin-like cysteine rich region | 4 | 8 | 1.94 | 0.00036 |
| PF01030 | Receptor L domain | 4 | 8 | 1.94 | 0.00036 |
| PF00200 | Disintegrin | 5 | 25 | 1.55 | 0.00047 |

**Table S14** Prediction the secretion ability of salivary protein in *D. ferox* genome by SignalP.

| **SignalP-5.0** | **Power** | **Description** |
| --- | --- | --- |
| TRINITY_DN210_c0_g1_i2.p1 | 0.901375 | CS pos: 21-22. IHG-LT. Pr: 0.7034 |
| TRINITY_DN40029_c2_g1_i2.p1 | 0.999144 | CS pos: 20-21. CFA-DV. Pr: 0.9757 |
| TRINITY_DN405_c0_g3_i1.p1 | 0.631949 | CS pos: 25-26. TIA-GR. Pr: 0.1458 |
| TRINITY_DN137_c0_g1_i7.p1 | 0.994351 | CS pos: 24-25. GDC-QG. Pr: 0.7690 |
| TRINITY_DN357_c1_g1_i7.p1 | 0.999244 | CS pos: 22-23. IQA-HL. Pr: 0.8037 |
| TRINITY_DN612_c0_g2_i1.p1 | 0.917406 | CS pos: 23-24. VRG-QL. Pr: 0.4582 |
| TRINITY_DN1103_c0_g1_i1.p1 | 0.967224 | CS pos: 18-19. VAP-CQ. Pr: 0.3292 |
| TRINITY_DN527_c0_g2_i2.p1 | 0.958922 | CS pos: 27-28. GHA-AV. Pr: 0.8685 |
| TRINITY_DN1027_c0_g1_i8.p1 | 0.867126 | CS pos: 26-27. SSA-GS. Pr: 0.2787 |
| TRINITY_DN394_c0_g1_i8.p1 | 0.911121 | CS pos: 25-26. LDG-NQ. Pr: 0.5141 |
| TRINITY_DN3739_c0_g3_i3.p1 | 0.7991 | CS pos: 18-19. VSG-QL. Pr: 0.7000 |
| TRINITY_DN3587_c0_g1_i1.p1 | 0.946146 | CS pos: 17-18. AIG-SL. Pr: 0.3071 |
| TRINITY_DN634_c0_g1_i2.p1 | 0.95835 | CS pos: 24-25. SRA-TE. Pr: 0.8802 |
| TRINITY_DN59_c0_g1_i10.p1 | 0.998587 | CS pos: 20-21. ANC-VF. Pr: 0.5262 |
| TRINITY_DN10423_c2_g1_i1.p1 | 0.999312 | CS pos: 20-21. TEA-SQ. Pr: 0.9603 |
| TRINITY_DN5297_c0_g1_i1.p1 | 0.91543 | CS pos: 36-37. AEA-AI. Pr: 0.5591 |
| TRINITY_DN992_c0_g1_i7.p1 | 0.967737 | CS pos: 20-21. CLS-FT. Pr: 0.7309 |
| TRINITY_DN1574_c0_g1_i2.p1 | 0.999124 | CS pos: 20-21. IMG-FD. Pr: 0.9510 |
| TRINITY_DN625_c1_g1_i1.p1 | 0.997032 | CS pos: 19-20. GEG-FF. Pr: 0.7368 |
| TRINITY_DN243_c0_g2_i1.p1 | 0.999191 | CS pos: 15-16. AVA-LP. Pr: 0.7181 |
| TRINITY_DN4402_c2_g1_i1.p1 | 0.995631 | CS pos: 26-27. SHA-FP. Pr: 0.8943 |
| TRINITY_DN10619_c0_g1_i2.p1 | 0.996659 | CS pos: 23-24. CEA-LE. Pr: 0.8517 |
| TRINITY_DN1848_c0_g1_i1.p1 | 0.945151 | CS pos: 20-21. ASG-AV. Pr: 0.3893 |
| TRINITY_DN243_c0_g1_i3.p1 | 0.996836 | CS pos: 15-16. AVA-FP. Pr: 0.7501 |
| TRINITY_DN4286_c0_g1_i1.p2 | 0.999597 | CS pos: 17-18. SFA-QN. Pr: 0.9200 |
| TRINITY_DN5866_c0_g2_i2.p1 | 0.668611 | CS pos: 41-42. CLS-QR. Pr: 0.5562 |
| TRINITY_DN3291_c0_g1_i8.p1 | 0.868967 | CS pos: 39-40. AQA-DR. Pr: 0.4788 |
| TRINITY_DN20629_c0_g1_i1.p1 | 0.994504 | CS pos: 22-23. VGA-ST. Pr: 0.9243 |
| TRINITY_DN95_c2_g1_i1.p1 | 0.855574 | CS pos: 33-34. ILG-QI. Pr: 0.5851 |
| TRINITY_DN24095_c2_g1_i1.p3 | 0.979763 | CS pos: 20-21. ALC-TE. Pr: 0.6379 |
| TRINITY_DN952_c0_g1_i1.p1 | 0.99961 | CS pos: 18-19. VTS-SE. Pr: 0.5889 |
| TRINITY_DN6545_c0_g3_i1.p1 | 0.971656 | CS pos: 23-24. GEA-QH. Pr: 0.6828 |
| TRINITY_DN33025_c0_g1_i1.p1 | 0.559671 | CS pos: 37-38. TFS-LS. Pr: 0.2904 |
| TRINITY_DN12538_c0_g2_i3.p2 | 0.994244 | CS pos: 27-28. AFA-EE. Pr: 0.9191 |
| TRINITY_DN1403_c0_g1_i2.p1 | 0.999393 | CS pos: 23-24. GQC-LE. Pr: 0.9294 |
| TRINITY_DN724_c0_g3_i1.p1 | 0.999494 | CS pos: 20-21. INA-KE. Pr: 0.9915 |
| TRINITY_DN183_c0_g1_i1.p1 | 0.999621 | CS pos: 18-19. GQC-LE. Pr: 0.9209 |
| TRINITY_DN2814_c1_g1_i1.p1 | 0.99519 | CS pos: 24-25. GVC-QG. Pr: 0.8349 |
| TRINITY_DN5380_c0_g1_i1.p2 | 0.999393 | CS pos: 21-22. TEA-SQ. Pr: 0.9593 |
| Dferox0G015880.1.p1 | 0.97258 | CS pos: 20-21. AGA-SF. Pr: 0.4911 |
| Dferox0G018510.1.p1 | 0.996894 | CS pos: 19-20. LSA-QR. Pr: 0.5677 |
| Dferox01G001190.1.p1 | 0.999384 | CS pos: 23-24. AQA-AR. Pr: 0.7151 |
| Dferox01G007020.1.p1 | 0.989495 | CS pos: 21-22. VKT-EC. Pr: 0.6421 |
| Dferox01G011320.1.p1 | 0.989784 | CS pos: 16-17. TGA-DH. Pr: 0.8986 |
| Dferox01G019120.1.p1 | 0.981549 | CS pos: 22-23. TRG-MD. Pr: 0.6312 |
| Dferox01G019330.1.p1 | 0.999132 | CS pos: 26-27. AFA-QE. Pr: 0.9791 |
| MSTRG.55.740.p17 | 0.999797 | CS pos: 18-19. VES-AI. Pr: 0.8472 |
| Dferox01G021200.1.p1 | 0.998596 | CS pos: 21-22. VDG-EY. Pr: 0.8963 |
| MSTRG.55.1314.p2 | 0.993357 | CS pos: 18-19. CLA-GV. Pr: 0.6383 |
| Dferox01G023940.1.p1 | 0.563452 | CS pos: 13-14. GSC-PN. Pr: 0.2127 |
| Dferox01G024600.1.p1 | 0.877714 | CS pos: 17-18. IRG-EL. Pr: 0.7600 |
| Dferox01G024610.1.p1 | 0.905052 | CS pos: 17-18. IRG-EL. Pr: 0.7890 |
| Dferox01G025450.1.p1 | 0.997196 | CS pos: 26-27. TSA-DT. Pr: 0.8832 |
| Dferox02G003860.1.p1 | 0.982009 | CS pos: 21-22. FQA-ST. Pr: 0.4494 |
| Dferox02G004850.1.p1 | 0.871126 | CS pos: 18-19. GCA-QV. Pr: 0.4869 |
| MSTRG.457.884.p3 | 0.902676 | CS pos: 25-26. TSA-GH. Pr: 0.4422 |
| MSTRG.457.908.p7 | 0.994327 | CS pos: 18-19. VFA-KH. Pr: 0.9569 |
| MSTRG.457.935.p1 | 0.993885 | CS pos: 19-20. LTA-QP. Pr: 0.3873 |
| MSTRG.457.961.p2 | 0.984739 | CS pos: 18-19. ILA-QK. Pr: 0.9432 |
| MSTRG.457.266.p31 | 0.997257 | CS pos: 16-17. ILA-GT. Pr: 0.3518 |
| MSTRG.457.267.p47 | 0.983465 | CS pos: 16-17. IHG-SH. Pr: 0.8783 |
| MSTRG.457.1376.p4 | 0.970208 | CS pos: 17-18. IMA-EH. Pr: 0.8383 |
| MSTRG.457.1405.p2 | 0.782 | CS pos: 24-25. SWT-KE. Pr: 0.7091 |
| Dferox02G017510.1.p1 | 0.51114 | CS pos: 50-51. VEA-QE. Pr: 0.4892 |
| MSTRG.457.1663.p1 | 0.997633 | CS pos: 20-21. GNG-DK. Pr: 0.9580 |
| Dferox03G001870.1.p1 | 0.992673 | CS pos: 17-18. ALS-QP. Pr: 0.8207 |
| Dferox03G002090.1.p1 | 0.995439 | CS pos: 17-18. ALS-QS. Pr: 0.4810 |
| Dferox03G002280.1.p1 | 0.993281 | CS pos: 17-18. AAG-SQ. Pr: 0.4080 |
| Dferox03G002450.1.p1 | 0.946733 | CS pos: 26-27. CSA-GS. Pr: 0.4102 |
| Dferox03G002890.1.p1 | 0.823009 | CS pos: 22-23. VTP-RE. Pr: 0.4838 |
| Dferox03G003460.1.p1 | 0.841975 | CS pos: 21-22. SFS-VK. Pr: 0.3932 |
| MSTRG.884.244.p1 | 0.987229 | CS pos: 18-19. VCC-SN. Pr: 0.5784 |
| MSTRG.884.324.p1 | 0.967605 | CS pos: 21-22. SLP-EK. Pr: 0.4286 |
| MSTRG.884.339.p7 | 0.998007 | CS pos: 19-20. SFG-QM. Pr: 0.8494 |
| Dferox03G005240.1.p1 | 0.99943 | CS pos: 18-19. SSG-DE. Pr: 0.6018 |
| MSTRG.890.175.p31 | 0.991926 | CS pos: 20-21. VYC-DV. Pr: 0.8298 |
| MSTRG.890.461.p3 | 0.974053 | CS pos: 18-19. SNC-QN. Pr: 0.8698 |
| MSTRG.890.485.p6 | 0.999614 | CS pos: 18-19. VTS-SE. Pr: 0.5893 |
| Dferox03G008550.1.p1 | 0.999514 | CS pos: 19-20. IYS-AC. Pr: 0.9292 |
| Dferox03G010040.1.p1 | 0.993257 | CS pos: 20-21. ATC-KP. Pr: 0.5847 |
| MSTRG.884.265.p22 | 0.999443 | CS pos: 17-18. AHA-DQ. Pr: 0.9442 |
| MSTRG.890.625.p1 | 0.984373 | CS pos: 18-19. SNC-QN. Pr: 0.8809 |
| MSTRG.890.650.p10 | 0.999268 | CS pos: 19-20. AEN-RP. Pr: 0.3822 |
| MSTRG.884.749.p4 | 0.999824 | CS pos: 18-19. GQC-LE. Pr: 0.9488 |
| MSTRG.890.263.p29 | 0.9891 | CS pos: 32-33. SDA-AN. Pr: 0.8901 |
| MSTRG.890.680.p3 | 0.99964 | CS pos: 19-20. AES-IP. Pr: 0.8479 |
| MSTRG.890.702.p3 | 0.9891 | CS pos: 32-33. SDA-AN. Pr: 0.8901 |
| Dferox03G012350.1.p1 | 0.971671 | CS pos: 24-25. TEA-AP. Pr: 0.9022 |
| Dferox03G012540.1.p1 | 0.946067 | CS pos: 19-20. ILA-DD. Pr: 0.8072 |
| MSTRG.884.928.p1 | 0.938809 | CS pos: 28-29. VHC-SS. Pr: 0.6748 |
| Dferox03G014670.1.p1 | 0.703749 | CS pos: 31-32. VKC-GD. Pr: 0.4769 |
| MSTRG.884.968.p7 | 0.999696 | CS pos: 24-25. SHA-QG. Pr: 0.9024 |
| Dferox03G015510.1.p1 | 0.998752 | CS pos: 19-20. VTS-DE. Pr: 0.8951 |
| Dferox03G015520.1.p1 | 0.998257 | CS pos: 21-22. TSG-EQ. Pr: 0.7426 |
| MSTRG.884.1057.p2 | 0.999557 | CS pos: 24-25. VSG-EE. Pr: 0.7707 |
| MSTRG.890.1037.p2 | 0.999649 | CS pos: 18-19. INA-EE. Pr: 0.9437 |
| Dferox04G002850.1.p1 | 0.998733 | CS pos: 18-19. IFC-FP. Pr: 0.5596 |
| MSTRG.1505.171.p2 | 0.999316 | CS pos: 17-18. SLG-AV. Pr: 0.5623 |
| Dferox04G002940.1.p1 | 0.995314 | CS pos: 20-21. TDS-RS. Pr: 0.9394 |
| Dferox04G006840.1.p1 | 0.999332 | CS pos: 24-25. GQA-QK. Pr: 0.8833 |
| Dferox04G006860.1.p1 | 0.701283 | CS pos: 39-40. CAA-ND. Pr: 0.4748 |
| Dferox04G007450.1.p1 | 0.982684 | CS pos: 21-22. AEA-EI. Pr: 0.8918 |
| MSTRG.1505.278.p12 | 0.985431 | CS pos: 19-20. SSG-FD. Pr: 0.5749 |
| MSTRG.2042.6.p6 | 0.938222 | CS pos: 25-26. VGC-VS. Pr: 0.6462 |
| MSTRG.2029.33.p5 | 0.990881 | CS pos: 19-20. IQS-HP. Pr: 0.6905 |
| MSTRG.2042.14.p4 | 0.998349 | CS pos: 20-21. ANC-VF. Pr: 0.5196 |
| MSTRG.2029.56.p3 | 0.989534 | CS pos: 20-21. GGC-RD. Pr: 0.6123 |
| MSTRG.2029.67.p1 | 0.999406 | CS pos: 22-23. IQA-HL. Pr: 0.8546 |
| Dferox05G001600.1.p1 | 0.82416 | CS pos: 20-21. CLG-IS. Pr: 0.4630 |
| Dferox05G001680.1.p1 | 0.99673 | CS pos: 18-19. IQA-ET. Pr: 0.9524 |
| Dferox05G001740.1.p1 | 0.963759 | CS pos: 20-21. CFS-FT. Pr: 0.6853 |
| MSTRG.2042.60.p1 | 0.997344 | CS pos: 18-19. SQA-NR. Pr: 0.9187 |
| MSTRG.2029.106.p1 | 0.994183 | CS pos: 23-24. VTG-WP. Pr: 0.4830 |
| MSTRG.2042.67.p1 | 0.998613 | CS pos: 20-21. ANC-VF. Pr: 0.5249 |
| MSTRG.2042.73.p7 | 0.988233 | CS pos: 20-21. GGC-RD. Pr: 0.6219 |
| MSTRG.2042.85.p2 | 0.983864 | CS pos: 19-20. TFA-GN. Pr: 0.9266 |
| MSTRG.2042.163.p3 | 0.999201 | CS pos: 20-21. VSA-QI. Pr: 0.6960 |
| MSTRG.2238.1.p1 | 0.999841 | CS pos: 21-22. SES-WG. Pr: 0.5757 |
| Dferox05G005970.1.p1 | 0.702977 | CS pos: 30-31. ALC-AK. Pr: 0.3091 |
| Dferox05G006280.1.p1 | 0.967808 | CS pos: 27-28. CMA-LE. Pr: 0.8941 |
| Dferox05G010650.1.p1 | 0.777584 | CS pos: 41-42. AKA-KE. Pr: 0.6714 |
| MSTRG.2029.850.p5 | 0.788579 | CS pos: 26-27. ALC-TE. Pr: 0.4704 |
| Dferox05G016570.1.p1 | 0.916277 | CS pos: 17-18. TSG-GS. Pr: 0.3369 |
| MSTRG.2702.2.p1 | 0.968165 | CS pos: 18-19. VAP-CQ. Pr: 0.3293 |
| MSTRG.2618.186.p1 | 0.918235 | CS pos: 27-28. ADG-AQ. Pr: 0.6676 |
| MSTRG.2618.282.p2 | 0.963215 | CS pos: 22-23. SSG-NE. Pr: 0.8273 |
| Dferox06G005870.1.p1 | 0.969957 | CS pos: 24-25. VDG-TC. Pr: 0.9172 |
| Dferox06G007460.1.p1 | 0.8405 | CS pos: 20-21. SYE-QK. Pr: 0.3690 |
| MSTRG.2618.514.p2 | 0.980646 | CS pos: 23-24. GQS-FV. Pr: 0.6535 |
| MSTRG.2661.583.p3 | 0.991373 | CS pos: 22-23. AQA-RV. Pr: 0.9242 |
| MSTRG.3253.26.p2 | 0.97857 | CS pos: 23-24. SRG-NS. Pr: 0.7754 |
| MSTRG.3253.40.p1 | 0.977425 | CS pos: 23-24. SRG-SE. Pr: 0.7434 |
| Dferox07G001770.1.p1 | 0.947785 | CS pos: 23-24. IHG-IT. Pr: 0.8160 |
| MSTRG.3321.1.p1 | 0.989819 | CS pos: 23-24. IHG-VA. Pr: 0.8113 |
| Dferox07G001850.1.p1 | 0.95174 | CS pos: 22-23. IHG-VT. Pr: 0.7809 |
| MSTRG.3247.141.p1 | 0.833319 | CS pos: 20-21. SLS-FD. Pr: 0.6915 |
| MSTRG.3336.1.p1 | 0.999157 | CS pos: 18-19. SHA-VN. Pr: 0.9703 |
| MSTRG.3358.1.p1 | 0.808846 | CS pos: 23-24. ICS-LN. Pr: 0.4061 |
| Dferox07G002870.1.p1 | 0.997177 | CS pos: 20-21. VKG-GI. Pr: 0.9072 |
| Dferox07G002910.1.p1 | 0.909846 | CS pos: 28-29. VKC-SE. Pr: 0.8483 |
| MSTRG.3253.100.p1 | 0.997066 | CS pos: 20-21. VKG-GI. Pr: 0.9075 |
| Dferox07G003190.1.p1 | 0.9839 | CS pos: 19-20. SLG-QT. Pr: 0.8147 |
| Dferox07G003250.1.p1 | 0.997435 | CS pos: 19-20. CCC-QS. Pr: 0.3407 |
| MSTRG.3247.298.p2 | 0.792927 | CS pos: 22-23. VRG-TH. Pr: 0.5568 |
| Dferox07G005240.1.p1 | 0.951629 | CS pos: 20-21. VDS-GV. Pr: 0.5443 |
| Dferox07G005570.1.p1 | 0.917868 | CS pos: 23-24. SYA-AV. Pr: 0.6720 |
| Dferox07G005590.1.p1 | 0.990096 | CS pos: 25-26. GHA-AV. Pr: 0.9068 |
| Dferox07G005610.1.p1 | 0.927638 | CS pos: 23-24. ASS-TV. Pr: 0.4127 |
| Dferox07G005710.1.p1 | 0.92878 | CS pos: 21-22. SEQ-TI. Pr: 0.5498 |
| Dferox07G005740.1.p1 | 0.987853 | CS pos: 18-19. LCC-DD. Pr: 0.7280 |
| Dferox07G005750.1.p1 | 0.991277 | CS pos: 25-26. VES-KS. Pr: 0.7845 |
| MSTRG.3247.641.p1 | 0.987508 | CS pos: 21-22. CFS-SP. Pr: 0.8178 |
| MSTRG.3631.7.p1 | 0.976617 | CS pos: 23-24. AVG-TK. Pr: 0.5183 |
| MSTRG.3856.13.p1 | 0.968173 | CS pos: 16-17. AAG-FR. Pr: 0.8358 |
| MSTRG.3856.63.p1 | 0.931663 | CS pos: 20-21. SFG-EG. Pr: 0.3549 |
| MSTRG.3856.68.p2 | 0.967603 | CS pos: 20-21. SFG-EG. Pr: 0.3387 |
| MSTRG.3856.70.p1 | 0.672819 | CS pos: 18-19. SFG-EG. Pr: 0.3204 |
| MSTRG.3856.76.p1 | 0.980234 | CS pos: 20-21. SFG-DG. Pr: 0.6655 |
| MSTRG.3856.79.p1 | 0.968545 | CS pos: 20-21. SFG-EG. Pr: 0.3168 |
| MSTRG.3856.82.p1 | 0.984896 | CS pos: 20-21. SFG-EG. Pr: 0.3263 |
| MSTRG.3885.62.p1 | 0.995771 | CS pos: 24-25. GDC-QG. Pr: 0.7771 |
| MSTRG.3885.60.p1 | 0.996069 | CS pos: 24-25. GDC-QG. Pr: 0.8994 |
| MSTRG.3885.65.p1 | 0.996069 | CS pos: 24-25. GDC-QG. Pr: 0.8994 |
| Dferox08G001770.1.p1 | 0.697005 | CS pos: 35-36. GVC-QG. Pr: 0.5740 |
| Dferox08G004020.1.p1 | 0.870936 | CS pos: 19-20. IKG-EV. Pr: 0.6298 |
| MSTRG.3885.192.p2 | 0.938486 | CS pos: 19-20. IIG-RD. Pr: 0.3588 |
| Dferox08G013210.1.p1 | 0.9937 | CS pos: 19-20. CTG-SS. Pr: 0.3699 |
| Dferox09G001010.1.p1 | 0.998729 | CS pos: 16-17. VSS-VE. Pr: 0.8332 |
| MSTRG.4647.115.p1 | 0.968381 | CS pos: 25-26. SDG-NE. Pr: 0.7812 |
| MSTRG.4647.141.p46 | 0.859531 | CS pos: 19-20. ARG-CG. Pr: 0.5186 |
| MSTRG.4647.141.p48 | 0.897992 | CS pos: 17-18. VVA-RG. Pr: 0.5721 |
| MSTRG.4647.151.p1 | 0.998333 | CS pos: 24-25. GGA-TN. Pr: 0.7689 |
| Dferox09G001630.1.p1 | 0.99794 | CS pos: 25-26. GGA-VT. Pr: 0.9228 |
| Dferox09G003100.1.p2 | 0.999362 | CS pos: 19-20. IRA-GI. Pr: 0.9066 |
| MSTRG.4647.287.p1 | 0.925451 | CS pos: 29-30. GEG-FF. Pr: 0.4797 |
| Dferox09G004610.1.p1 | 0.995786 | CS pos: 22-23. GRA-EC. Pr: 0.5048 |
| Dferox09G005390.1.p1 | 0.961159 | CS pos: 22-23. ITG-DY. Pr: 0.4865 |
| Dferox09G007480.1.p1 | 0.902935 | CS pos: 17-18. VGA-AL. Pr: 0.3297 |
| MSTRG.4646.430.p1 | 0.998895 | CS pos: 23-24. SAA-LV. Pr: 0.5312 |
| MSTRG.4647.566.p2 | 0.797176 | CS pos: 18-19. VSC-SI. Pr: 0.5338 |
| MSTRG.5052.1.p2 | 0.999737 | CS pos: 19-20. LQT-SE. Pr: 0.2583 |
| Dferox09G011080.1.p1 | 0.977532 | CS pos: 27-28. TSS-DL. Pr: 0.7981 |
| MSTRG.4646.685.p2 | 0.598901 | CS pos: 25-26. TIA-GR. Pr: 0.1315 |
| MSTRG.4646.419.p11 | 0.821116 | CS pos: 17-18. ALA-SI. Pr: 0.7616 |
| Dferox09G015710.1.p1 | 0.829232 | CS pos: 17-18. ALA-MI. Pr: 0.2945 |
| MSTRG.5281.1.p1 | 0.998186 | CS pos: 23-24. ALC-FL. Pr: 0.8902 |
| Dferox0G013680.1.p1 | 0.98856 | CS pos: 19-20. CTA-QT. Pr: 0.9222 |

**Table S15** Prediction the secretion ability of salivary protein in *D. ferox* genome by TargetP.

| **TargetP-2.0** | **Power** | **Description** |
| --- | --- | --- |
| TRINITY_DN210_c0_g1_i2.p1 | 0.99538 | CS pos: 21-22. IHG-LT. Pr: 0.8859 |
| TRINITY_DN40029_c2_g1_i2.p1 | 0.99988 | CS pos: 20-21. CFA-DV. Pr: 0.9506 |
| TRINITY_DN405_c0_g3_i1.p1 | 0.85655 | CS pos: 25-26. TIA-GR. Pr: 0.4105 |
| TRINITY_DN137_c0_g1_i7.p1 | 0.998806 | CS pos: 24-25. GDC-QG. Pr: 0.9509 |
| TRINITY_DN357_c1_g1_i7.p1 | 0.999982 | CS pos: 22-23. IQA-HL. Pr: 0.7576 |
| TRINITY_DN612_c0_g2_i1.p1 | 0.998202 | CS pos: 23-24. VRG-QL. Pr: 0.6297 |
| TRINITY_DN1103_c0_g1_i1.p1 | 0.997562 | CS pos: 19-20. APC-QV. Pr: 0.6080 |
| TRINITY_DN527_c0_g2_i2.p1 | 0.988478 | CS pos: 27-28. GHA-AV. Pr: 0.9496 |
| TRINITY_DN1027_c0_g1_i8.p1 | 0.987502 | CS pos: 26-27. SSA-GS. Pr: 0.4647 |
| TRINITY_DN394_c0_g1_i8.p1 | 0.986042 | CS pos: 25-26. LDG-NQ. Pr: 0.8755 |
| TRINITY_DN3739_c0_g3_i3.p1 | 0.957423 | CS pos: 18-19. VSG-QL. Pr: 0.9029 |
| TRINITY_DN3587_c0_g1_i1.p1 | 0.999419 | CS pos: 18-19. IGS-LD. Pr: 0.2926 |
| TRINITY_DN634_c0_g1_i2.p1 | 0.999302 | CS pos: 24-25. SRA-TE. Pr: 0.9210 |
| TRINITY_DN59_c0_g1_i10.p1 | 0.999975 | CS pos: 20-21. ANC-VF. Pr: 0.6745 |
| TRINITY_DN10423_c2_g1_i1.p1 | 0.999493 | CS pos: 20-21. TEA-SQ. Pr: 0.8468 |
| TRINITY_DN5297_c0_g1_i1.p1 | 0.998103 | CS pos: 36-37. AEA-AI. Pr: 0.6675 |
| TRINITY_DN992_c0_g1_i7.p1 | 0.998541 | CS pos: 20-21. CLS-FT. Pr: 0.7508 |
| TRINITY_DN1574_c0_g1_i2.p1 | 0.999979 | CS pos: 20-21. IMG-FD. Pr: 0.9660 |
| TRINITY_DN139_c0_g1_i1.p5 | 0.541907 | CS pos: 20-21. NLS-WV. Pr: 0.1353 |
| TRINITY_DN625_c1_g1_i1.p1 | 0.999955 | CS pos: 19-20. GEG-FF. Pr: 0.8015 |
| TRINITY_DN243_c0_g2_i1.p1 | 0.99998 | CS pos: 15-16. AVA-LP. Pr: 0.8646 |
| TRINITY_DN4402_c2_g1_i1.p1 | 0.999744 | CS pos: 26-27. SHA-FP. Pr: 0.9013 |
| TRINITY_DN10619_c0_g1_i2.p1 | 0.999784 | CS pos: 23-24. CEA-LE. Pr: 0.8041 |
| TRINITY_DN1848_c0_g1_i1.p1 | 0.981488 | CS pos: 20-21. ASG-AV. Pr: 0.3156 |
| TRINITY_DN243_c0_g1_i3.p1 | 0.999944 | CS pos: 15-16. AVA-FP. Pr: 0.8707 |
| TRINITY_DN4286_c0_g1_i1.p2 | 0.999996 | CS pos: 17-18. SFA-QN. Pr: 0.9131 |
| TRINITY_DN5866_c0_g2_i2.p1 | 0.976509 | CS pos: 41-42. CLS-QR. Pr: 0.9267 |
| TRINITY_DN3291_c0_g1_i8.p1 | 0.982156 | CS pos: 37-38. INA-QA. Pr: 0.6535 |
| TRINITY_DN20629_c0_g1_i1.p1 | 0.994625 | CS pos: 22-23. VGA-ST. Pr: 0.8796 |
| TRINITY_DN95_c2_g1_i1.p1 | 0.999178 | CS pos: 33-34. ILG-QI. Pr: 0.8760 |
| TRINITY_DN24095_c2_g1_i1.p3 | 0.999675 | CS pos: 20-21. ALC-TE. Pr: 0.8628 |
| TRINITY_DN952_c0_g1_i1.p1 | 0.999998 | CS pos: 18-19. VTS-SE. Pr: 0.8354 |
| TRINITY_DN6545_c0_g3_i1.p1 | 0.993601 | CS pos: 23-24. GEA-QH. Pr: 0.8191 |
| TRINITY_DN33025_c0_g1_i1.p1 | 0.880156 | CS pos: 37-38. TFS-LS. Pr: 0.6096 |
| TRINITY_DN12538_c0_g2_i3.p2 | 0.999825 | CS pos: 27-28. AFA-EE. Pr: 0.9686 |
| TRINITY_DN231_c0_g1_i1.p1 | 0.701464 | CS pos: 29-30. ARG-SS. Pr: 0.8026 |
| TRINITY_DN1403_c0_g1_i2.p1 | 0.999977 | CS pos: 23-24. GQC-LE. Pr: 0.9577 |
| TRINITY_DN724_c0_g3_i1.p1 | 0.999989 | CS pos: 20-21. INA-KE. Pr: 0.9873 |
| TRINITY_DN183_c0_g1_i1.p1 | 1 | CS pos: 18-19. GQC-LE. Pr: 0.9591 |
| TRINITY_DN2814_c1_g1_i1.p1 | 0.999619 | CS pos: 24-25. GVC-QG. Pr: 0.8861 |
| TRINITY_DN4843_c0_g1_i1.p4 | 0.973576 | CS pos: 14-15. IQA-HL. Pr: 0.9577 |
| TRINITY_DN5380_c0_g1_i1.p2 | 0.999995 | CS pos: 21-22. TEA-SQ. Pr: 0.8994 |
| TRINITY_DN5324_c0_g1_i2.p1 | 0.748064 | CS pos: 27-28. VSG-IF. Pr: 0.5626 |
| Dferox0G015880.1.p1 | 0.993609 | CS pos: 25-26. VGC-KD. Pr: 0.3247 |
| Dferox0G018510.1.p1 | 0.999228 | CS pos: 18-19. SLS-AQ. Pr: 0.4730 |
| Dferox01G001190.1.p1 | 0.999968 | CS pos: 21-22. INA-QA. Pr: 0.5970 |
| Dferox01G007020.1.p1 | 0.995493 | CS pos: 21-22. VKT-EC. Pr: 0.8665 |
| Dferox01G011320.1.p1 | 0.999918 | CS pos: 16-17. TGA-DH. Pr: 0.8803 |
| Dferox01G019120.1.p1 | 0.99977 | CS pos: 22-23. TRG-MD. Pr: 0.8537 |
| Dferox01G019330.1.p1 | 0.999958 | CS pos: 26-27. AFA-QE. Pr: 0.9379 |
| MSTRG.55.740.p17 | 0.999998 | CS pos: 18-19. VES-AI. Pr: 0.5776 |
| Dferox01G021200.1.p1 | 0.999914 | CS pos: 21-22. VDG-EY. Pr: 0.9770 |
| MSTRG.55.1314.p2 | 0.999941 | CS pos: 18-19. CLA-GV. Pr: 0.8313 |
| Dferox01G023940.1.p1 | 0.930985 | CS pos: 14-15. SCP-ND. Pr: 0.6041 |
| Dferox01G024600.1.p1 | 0.991225 | CS pos: 17-18. IRG-EL. Pr: 0.9616 |
| Dferox01G024610.1.p1 | 0.985151 | CS pos: 17-18. IRG-EL. Pr: 0.9601 |
| Dferox01G025450.1.p1 | 0.999916 | CS pos: 26-27. TSA-DT. Pr: 0.8831 |
| Dferox02G003860.1.p1 | 0.979526 | CS pos: 24-25. STG-FK. Pr: 0.4672 |
| Dferox02G004850.1.p1 | 0.998352 | CS pos: 18-19. GCA-QV. Pr: 0.6315 |
| MSTRG.457.884.p3 | 0.983336 | CS pos: 25-26. TSA-GH. Pr: 0.5569 |
| MSTRG.457.908.p7 | 0.99968 | CS pos: 18-19. VFA-KH. Pr: 0.9642 |
| MSTRG.457.935.p1 | 0.999905 | CS pos: 21-22. AQP-GC. Pr: 0.3474 |
| MSTRG.457.961.p2 | 0.996511 | CS pos: 18-19. ILA-QK. Pr: 0.9668 |
| MSTRG.457.266.p31 | 0.99953 | CS pos: 16-17. ILA-GT. Pr: 0.7628 |
| MSTRG.457.1290.p1 | 0.763374 | CS pos: 30-31. VWS-QV. Pr: 0.6778 |
| MSTRG.457.267.p47 | 0.999825 | CS pos: 16-17. IHG-SH. Pr: 0.9378 |
| MSTRG.457.1376.p4 | 0.9982 | CS pos: 17-18. IMA-EH. Pr: 0.9563 |
| MSTRG.457.1405.p2 | 0.99446 | CS pos: 24-25. SWT-KE. Pr: 0.9211 |
| Dferox02G017510.1.p1 | 0.641592 | CS pos: 50-51. VEA-QE. Pr: 0.8845 |
| MSTRG.457.1663.p1 | 0.999826 | CS pos: 20-21. GNG-DK. Pr: 0.9817 |
| Dferox03G001870.1.p1 | 0.999521 | CS pos: 17-18. ALS-QP. Pr: 0.8671 |
| Dferox03G002090.1.p1 | 0.999738 | CS pos: 17-18. ALS-QS. Pr: 0.6123 |
| Dferox03G002280.1.p1 | 0.999219 | CS pos: 17-18. AAG-SQ. Pr: 0.6294 |
| Dferox03G002450.1.p1 | 0.993662 | CS pos: 26-27. CSA-GS. Pr: 0.6515 |
| Dferox03G002880.1.p1 | 0.613291 | CS pos: 29-30. ATA-VF. Pr: 0.4503 |
| Dferox03G002890.1.p1 | 0.981761 | CS pos: 22-23. VTP-RE. Pr: 0.5371 |
| Dferox03G003460.1.p1 | 0.977968 | CS pos: 21-22. SFS-VK. Pr: 0.3758 |
| MSTRG.884.244.p1 | 0.995302 | CS pos: 18-19. VCC-SN. Pr: 0.5424 |
| MSTRG.884.324.p1 | 0.993192 | CS pos: 21-22. SLP-EK. Pr: 0.4197 |
| MSTRG.884.339.p7 | 0.999982 | CS pos: 19-20. SFG-QM. Pr: 0.8412 |
| Dferox03G005240.1.p1 | 0.999996 | CS pos: 18-19. SSG-DE. Pr: 0.8160 |
| MSTRG.890.175.p31 | 0.99985 | CS pos: 20-21. VYC-DV. Pr: 0.8007 |
| MSTRG.890.461.p3 | 0.997185 | CS pos: 18-19. SNC-QN. Pr: 0.9522 |
| MSTRG.890.485.p6 | 0.999998 | CS pos: 18-19. VTS-SE. Pr: 0.8353 |
| Dferox03G008550.1.p1 | 0.999994 | CS pos: 19-20. IYS-AC. Pr: 0.9335 |
| Dferox03G010040.1.p1 | 0.998238 | CS pos: 20-21. ATC-KP. Pr: 0.7345 |
| MSTRG.884.265.p22 | 0.999985 | CS pos: 17-18. AHA-DQ. Pr: 0.9421 |
| MSTRG.890.625.p1 | 0.997383 | CS pos: 18-19. SNC-QN. Pr: 0.9544 |
| MSTRG.890.650.p10 | 0.999997 | CS pos: 16-17. TFS-AE. Pr: 0.3807 |
| MSTRG.884.749.p4 | 0.999992 | CS pos: 18-19. GQC-LE. Pr: 0.9305 |
| MSTRG.890.263.p29 | 0.999875 | CS pos: 32-33. SDA-AN. Pr: 0.9232 |
| MSTRG.890.680.p3 | 0.999998 | CS pos: 19-20. AES-IP. Pr: 0.8773 |
| MSTRG.890.702.p3 | 0.999907 | CS pos: 32-33. SDA-AN. Pr: 0.9237 |
| Dferox03G012350.1.p1 | 0.996873 | CS pos: 24-25. TEA-AP. Pr: 0.8444 |
| Dferox03G012540.1.p1 | 0.99687 | CS pos: 19-20. ILA-DD. Pr: 0.8803 |
| Dferox03G014670.1.p1 | 0.959311 | CS pos: 31-32. VKC-GD. Pr: 0.9187 |
| MSTRG.884.968.p7 | 1 | CS pos: 24-25. SHA-QG. Pr: 0.9174 |
| Dferox03G015510.1.p1 | 0.999888 | CS pos: 19-20. VTS-DE. Pr: 0.9540 |
| Dferox03G015520.1.p1 | 0.998992 | CS pos: 21-22. TSG-EQ. Pr: 0.4376 |
| MSTRG.884.1057.p2 | 0.999998 | CS pos: 24-25. VSG-EE. Pr: 0.7408 |
| MSTRG.890.1037.p2 | 0.999799 | CS pos: 18-19. INA-EE. Pr: 0.9655 |
| Dferox04G002850.1.p1 | 0.999956 | CS pos: 18-19. IFC-FP. Pr: 0.6563 |
| MSTRG.1505.171.p2 | 0.999996 | CS pos: 17-18. SLG-AV. Pr: 0.4445 |
| Dferox04G002940.1.p1 | 0.99995 | CS pos: 20-21. TDS-RS. Pr: 0.9197 |
| Dferox04G006840.1.p1 | 0.999979 | CS pos: 24-25. GQA-QK. Pr: 0.7762 |
| Dferox04G006860.1.p1 | 0.984156 | CS pos: 39-40. CAA-ND. Pr: 0.6582 |
| Dferox04G007450.1.p1 | 0.999442 | CS pos: 21-22. AEA-EI. Pr: 0.8599 |
| MSTRG.1505.278.p12 | 0.99793 | CS pos: 19-20. SSG-FD. Pr: 0.7206 |
| MSTRG.2042.6.p6 | 0.99653 | CS pos: 25-26. VGC-VS. Pr: 0.7879 |
| MSTRG.2029.33.p5 | 0.998636 | CS pos: 19-20. IQS-HP. Pr: 0.7744 |
| MSTRG.2042.14.p4 | 0.999924 | CS pos: 20-21. ANC-VF. Pr: 0.6849 |
| MSTRG.2029.56.p3 | 0.999787 | CS pos: 20-21. GGC-RD. Pr: 0.7205 |
| MSTRG.2029.67.p1 | 0.999991 | CS pos: 22-23. IQA-HL. Pr: 0.8002 |
| Dferox05G001600.1.p1 | 0.918384 | CS pos: 20-21. CLG-IS. Pr: 0.5955 |
| Dferox05G001680.1.p1 | 0.999647 | CS pos: 18-19. IQA-ET. Pr: 0.9197 |
| Dferox05G001740.1.p1 | 0.998905 | CS pos: 20-21. CFS-FT. Pr: 0.7844 |
| MSTRG.2042.60.p1 | 0.999404 | CS pos: 18-19. SQA-NR. Pr: 0.8904 |
| MSTRG.2029.106.p1 | 0.999894 | CS pos: 18-19. CLS-TD. Pr: 0.4922 |
| MSTRG.2042.67.p1 | 0.999966 | CS pos: 20-21. ANC-VF. Pr: 0.6711 |
| MSTRG.2042.73.p7 | 0.999858 | CS pos: 20-21. GGC-RD. Pr: 0.7370 |
| MSTRG.2042.85.p2 | 0.997687 | CS pos: 19-20. TFA-GN. Pr: 0.8767 |
| MSTRG.2042.163.p3 | 0.999967 | CS pos: 20-21. VSA-QI. Pr: 0.8002 |
| MSTRG.2238.1.p1 | 0.999999 | CS pos: 21-22. SES-WG. Pr: 0.8870 |
| Dferox05G005970.1.p1 | 0.904365 | CS pos: 30-31. ALC-AK. Pr: 0.6658 |
| Dferox05G006280.1.p1 | 0.998826 | CS pos: 27-28. CMA-LE. Pr: 0.8807 |
| Dferox05G010650.1.p1 | 0.766836 | CS pos: 41-42. AKA-KE. Pr: 0.8814 |
| MSTRG.2029.850.p5 | 0.976828 | CS pos: 26-27. ALC-TE. Pr: 0.8388 |
| Dferox05G016570.1.p1 | 0.996729 | CS pos: 18-19. SGG-SS. Pr: 0.6354 |
| MSTRG.2702.2.p1 | 0.99749 | CS pos: 19-20. APC-QV. Pr: 0.6099 |
| MSTRG.2618.186.p1 | 0.960303 | CS pos: 27-28. ADG-AQ. Pr: 0.7327 |
| MSTRG.2618.282.p2 | 0.998058 | CS pos: 22-23. SSG-NE. Pr: 0.8936 |
| Dferox06G005870.1.p1 | 0.990434 | CS pos: 24-25. VDG-TC. Pr: 0.9777 |
| Dferox06G007460.1.p1 | 0.997866 | CS pos: 20-21. SYE-QK. Pr: 0.3845 |
| MSTRG.2618.514.p2 | 0.99586 | CS pos: 23-24. GQS-FV. Pr: 0.8810 |
| MSTRG.2661.583.p3 | 0.996156 | CS pos: 22-23. AQA-RV. Pr: 0.9434 |
| MSTRG.3253.26.p2 | 0.998971 | CS pos: 23-24. SRG-NS. Pr: 0.9544 |
| MSTRG.3253.40.p1 | 0.998862 | CS pos: 23-24. SRG-SE. Pr: 0.9410 |
| Dferox07G001770.1.p1 | 0.999375 | CS pos: 23-24. IHG-IT. Pr: 0.9492 |
| MSTRG.3321.1.p1 | 0.998921 | CS pos: 23-24. IHG-VA. Pr: 0.9230 |
| Dferox07G001850.1.p1 | 0.997876 | CS pos: 22-23. IHG-VT. Pr: 0.8820 |
| MSTRG.3247.141.p1 | 0.953985 | CS pos: 20-21. SLS-FD. Pr: 0.8566 |
| MSTRG.3336.1.p1 | 0.999972 | CS pos: 18-19. SHA-VN. Pr: 0.9709 |
| MSTRG.3358.1.p1 | 0.915721 | CS pos: 23-24. ICS-LN. Pr: 0.7435 |
| Dferox07G002870.1.p1 | 0.999433 | CS pos: 20-21. VKG-GI. Pr: 0.8692 |
| Dferox07G002910.1.p1 | 0.983163 | CS pos: 28-29. VKC-SE. Pr: 0.9385 |
| MSTRG.3253.100.p1 | 0.999469 | CS pos: 20-21. VKG-GI. Pr: 0.8693 |
| Dferox07G003190.1.p1 | 0.996734 | CS pos: 19-20. SLG-QT. Pr: 0.8737 |
| Dferox07G003250.1.p1 | 0.999304 | CS pos: 19-20. CCC-QS. Pr: 0.4708 |
| MSTRG.3247.298.p2 | 0.935144 | CS pos: 22-23. VRG-TH. Pr: 0.8678 |
| Dferox07G005240.1.p1 | 0.983097 | CS pos: 20-21. VDS-GV. Pr: 0.5450 |
| Dferox07G005570.1.p1 | 0.985231 | CS pos: 23-24. SYA-AV. Pr: 0.7962 |
| Dferox07G005590.1.p1 | 0.996411 | CS pos: 25-26. GHA-AV. Pr: 0.9497 |
| Dferox07G005610.1.p1 | 0.992473 | CS pos: 23-24. ASS-TV. Pr: 0.7143 |
| Dferox07G005710.1.p1 | 0.996753 | CS pos: 21-22. SEQ-TI. Pr: 0.7168 |
| Dferox07G005740.1.p1 | 0.998324 | CS pos: 17-18. NLC-CD. Pr: 0.4965 |
| Dferox07G005750.1.p1 | 0.998079 | CS pos: 25-26. VES-KS. Pr: 0.7475 |
| MSTRG.3247.641.p1 | 0.991567 | CS pos: 21-22. CFS-SP. Pr: 0.8412 |
| MSTRG.3631.7.p1 | 0.999496 | CS pos: 23-24. AVG-TK. Pr: 0.7221 |
| MSTRG.3856.13.p1 | 0.996475 | CS pos: 16-17. AAG-FR. Pr: 0.9103 |
| MSTRG.3856.63.p1 | 0.996636 | CS pos: 20-21. SFG-EG. Pr: 0.3591 |
| MSTRG.3856.68.p2 | 0.993273 | CS pos: 20-21. SFG-EG. Pr: 0.3584 |
| MSTRG.3856.70.p1 | 0.7759 | CS pos: 18-19. SFG-EG. Pr: 0.4059 |
| MSTRG.3856.76.p1 | 0.992175 | CS pos: 20-21. SFG-DG. Pr: 0.4801 |
| MSTRG.3856.79.p1 | 0.990885 | CS pos: 20-21. SFG-EG. Pr: 0.3317 |
| MSTRG.3856.82.p1 | 0.994983 | CS pos: 20-21. SFG-EG. Pr: 0.3467 |
| MSTRG.3885.62.p1 | 0.999448 | CS pos: 24-25. GDC-QG. Pr: 0.9501 |
| MSTRG.3885.60.p1 | 0.999754 | CS pos: 24-25. GDC-QG. Pr: 0.9268 |
| MSTRG.3885.65.p1 | 0.999717 | CS pos: 24-25. GDC-QG. Pr: 0.9268 |
| Dferox08G001770.1.p1 | 0.967958 | CS pos: 35-36. GVC-QG. Pr: 0.8681 |
| Dferox08G004020.1.p1 | 0.983131 | CS pos: 19-20. IKG-EV. Pr: 0.7318 |
| MSTRG.3885.192.p2 | 0.994956 | CS pos: 19-20. IIG-RD. Pr: 0.6211 |
| Dferox08G013210.1.p1 | 0.998521 | CS pos: 19-20. CTG-SS. Pr: 0.3687 |
| Dferox09G001010.1.p1 | 0.999977 | CS pos: 16-17. VSS-VE. Pr: 0.9119 |
| MSTRG.4647.115.p1 | 0.99333 | CS pos: 25-26. SDG-NE. Pr: 0.9222 |
| MSTRG.4647.141.p46 | 0.995777 | CS pos: 21-22. GCG-DD. Pr: 0.5322 |
| MSTRG.4647.141.p48 | 0.98849 | CS pos: 17-18. VVA-RG. Pr: 0.6356 |
| MSTRG.4647.151.p1 | 0.999991 | CS pos: 24-25. GGA-TN. Pr: 0.8699 |
| Dferox09G001630.1.p1 | 0.999376 | CS pos: 25-26. GGA-VT. Pr: 0.9730 |
| Dferox09G003100.1.p2 | 0.999906 | CS pos: 19-20. IRA-GI. Pr: 0.9171 |
| MSTRG.4647.287.p1 | 0.998548 | CS pos: 29-30. GEG-FF. Pr: 0.7600 |
| MSTRG.4647.320.p1 | 0.638216 | CS pos: 33-34. SEA-VN. Pr: 0.6999 |
| Dferox09G004610.1.p1 | 0.999914 | CS pos: 22-23. GRA-EC. Pr: 0.5690 |
| Dferox09G005390.1.p1 | 0.999567 | CS pos: 22-23. ITG-DY. Pr: 0.7906 |
| Dferox09G007480.1.p1 | 0.98778 | CS pos: 20-21. ALG-IG. Pr: 0.4781 |
| MSTRG.4646.430.p1 | 0.999972 | CS pos: 23-24. SAA-LV. Pr: 0.7888 |
| MSTRG.4647.566.p2 | 0.960434 | CS pos: 18-19. VSC-SI. Pr: 0.7553 |
| MSTRG.5052.1.p2 | 1 | CS pos: 18-19. SLQ-TS. Pr: 0.2955 |
| Dferox09G011080.1.p1 | 0.988251 | CS pos: 27-28. TSS-DL. Pr: 0.8289 |
| MSTRG.4646.685.p2 | 0.84085 | CS pos: 25-26. TIA-GR. Pr: 0.3802 |
| MSTRG.4646.419.p11 | 0.974516 | CS pos: 17-18. ALA-SI. Pr: 0.9611 |
| MSTRG.5242.1.p1 | 0.862016 | CS pos: 27-28. VFG-FN. Pr: 0.6895 |
| Dferox09G015710.1.p1 | 0.996154 | CS pos: 17-18. ALA-MI. Pr: 0.6320 |
| MSTRG.5281.1.p1 | 0.999999 | CS pos: 23-24. ALC-FL. Pr: 0.9651 |
| Dferox0G013680.1.p1 | 0.998338 | CS pos: 19-20. CTA-QT. Pr: 0.8604 |

**Table S16** Prediction the secretion ability of salivary protein in *D. ferox* genome by TMHMM.

| **Protein ID** | **Topology** |
| --- | --- |
| TRINITY_DN851_c4_g1_i2.p1 | o |
| TRINITY_DN263_c1_g2_i1.p1 | i7-29o |
| TRINITY_DN405_c0_g3_i1.p1 | i7-26o |
| TRINITY_DN6284_c0_g5_i1.p1 | i21-40o |
| TRINITY_DN4672_c2_g1_i3.p1 | o633-655i |
| TRINITY_DN8891_c0_g1_i21.p1 | i99-121o237-259i272-294o329-351i |
| TRINITY_DN13312_c0_g2_i7.p1 | i75-97o123-145i184-206o216-238i |
| TRINITY_DN1825_c0_g3_i2.p1 | i12-34o68-90i97-119o258-280i |
| TRINITY_DN3024_c0_g1_i1.p1 | o15-37i79-101o111-133i261-283o |
| TRINITY_DN1027_c0_g1_i8.p1 | i7-29o |
| TRINITY_DN7337_c0_g1_i1.p1 | i5-27o |
| TRINITY_DN13819_c0_g1_i4.p1 | i21-43o72-94i |
| TRINITY_DN1588_c0_g1_i1.p1 | o113-135i177-199o214-236i |
| TRINITY_DN255_c0_g1_i2.p1 | i12-34o54-76i83-105o195-217i |
| TRINITY_DN5297_c0_g1_i1.p1 | o10-32i |
| TRINITY_DN992_c0_g1_i7.p1 | i7-29o |
| TRINITY_DN8973_c0_g2_i1.p2 | i90-112o |
| TRINITY_DN10986_c0_g2_i1.p1 | i20-42o62-84i91-113o216-238i |
| TRINITY_DN4402_c2_g1_i1.p1 | i13-35o |
| TRINITY_DN3291_c0_g1_i8.p1 | o15-37i |
| TRINITY_DN24095_c2_g1_i1.p3 | i5-27o |
| TRINITY_DN12538_c0_g2_i3.p2 | i13-27o |
| TRINITY_DN1698_c0_g1_i3.p1 | i13-35o71-93i |
| Dferox01G021200.1.p1 | o2377-2396i2587-2606o2626-2648i2754-2776o2796-2818i2885-2907o3149-3167i3187-3209o3229-3251i3277-3299o3338-3360i |
| MSTRG.457.443.p1 | o266-288i |
| Dferox02G003860.1.p1 | i5-24o |
| MSTRG.457.629.p1 | o8344-8366i |
| MSTRG.458.712.p2 | o50-67i80-102o112-134i141-158o243-265i274-296o322-344i356-378o414-436i456-478o488-510i526-548o568-590i |
| MSTRG.457.1028.p4 | o10-32i52-71o81-103i110-127o151-173i |
| Dferox02G021080.1.p1 | i104-126o146-168i208-230o240-262i |
| Dferox03G002450.1.p1 | i13-32o |
| Dferox03G002880.1.p1 | i13-32o |
| Dferox03G002890.1.p1 | i7-26o |
| MSTRG.890.175.p31 | i7-29o |
| Dferox03G012340.1.p1 | i7-29o |
| Dferox03G012350.1.p1 | i7-29o |
| MSTRG.884.928.p1 | i5-27o131-153i173-195o457-479i491-513o804-826i |
| Dferox03G014670.1.p1 | i7-29o |
| MSTRG.884.968.p7 | i7-24o |
| MSTRG.884.1057.p2 | o5-24i |
| MSTRG.1505.27.p13 | o988-1010i1031-1053o1073-1090i1406-1423o1438-1460i1482-1504o1526-1548i1586-1608o1612-1626i1667-1689o |
| Dferox04G007740.1.p1 | o2401-2423i |
| MSTRG.2042.6.p6 | i12-31o |
| Dferox05G001740.1.p1 | i7-29o |
| Dferox05G005970.1.p1 | o15-37i |
| Dferox05G006280.1.p1 | i13-30o |
| Dferox05G010650.1.p1 | o557-574i |
| MSTRG.2029.850.p5 | i12-34o |
| Dferox05G012050.1.p1 | o5-27i114-136o163-185i192-214o224-246i |
| Dferox05G013010.1.p1 | i218-240o276-298i311-333o348-370i |
| Dferox05G013030.1.p1 | i7-29o67-89i96-118o133-155i |
| Dferox06G004890.1.p2 | o598-620i |
| MSTRG.2618.300.p1 | i13-35o80-102i109-131o161-180i262-284o |
| Dferox06G005870.1.p1 | o1403-1425i |
| Dferox06G007340.1.p1 | o5233-5255i |
| Dferox07G001770.1.p1 | i5-27o |
| Dferox07G003250.1.p1 | o624-646i |
| Dferox07G003770.1.p1 | o15-34i |
| Dferox07G005570.1.p1 | i7-29o |
| Dferox07G005750.1.p1 | i5-23o |
| MSTRG.3247.641.p1 | o585-607i |
| Dferox07G013720.1.p1 | o4021-4043i |
| MSTRG.3856.63.p1 | i5-27o |
| MSTRG.3856.68.p2 | i5-27o53-75i |
| MSTRG.3856.79.p1 | i5-27o54-76i |
| MSTRG.4647.141.p46 | i53-75o |
| MSTRG.4647.141.p48 | i57-79o |
| MSTRG.4647.151.p1 | o5-27i |
| Dferox09G001630.1.p1 | i106-128o |
| MSTRG.4825.1.p1 | i34-56o |
| MSTRG.4825.2.p1 | i44-66o |
| MSTRG.4647.287.p1 | i2-24o |
| MSTRG.4647.320.p1 | i17-39o |
| Dferox09G005390.1.p1 | i5-22o |
| Dferox09G007410.1.p1 | o260-282i295-317o322-344i372-394o417-439i452-474o |
| Dferox09G007480.1.p1 | i5-27o |
| MSTRG.4646.408.p1 | o4592-4614i |
| MSTRG.4646.408.p2 | i13-30o |
| MSTRG.4646.430.p1 | o5-27i |
| MSTRG.4646.66.p8 | i7-29o250-272i |
| MSTRG.4647.566.p2 | i5-27o |
| Dferox09G011080.1.p1 | i13-32o5511-5533i |
| MSTRG.4646.685.p2 | i7-26o |
| MSTRG.4646.419.p11 | i5-27o |
| Dferox09G015710.1.p1 | i7-29o |
| MSTRG.5281.1.p1 | o5-27i |
